# Supplementary material for: A cancer cell membrane coated, doxorubicin and microRNA co-encapsulated nanoplatform for colorectal cancer theranostics
Source: Mol Ther Oncolytics. 2022 Dec 7;28:182–96. doi: 10.1016/j.omto.2022.12.002 (PMC9937835; doi:10.1016/j.omto.2022.12.002)
Supplement: Document S1. Figures S1–S9 and Tables S1 and S2 [file mmc1.pdf]

OMTO, Volume *28*

Supplemental information

A cancer cell membrane coated, doxorubicin and microRNA co-encapsulated nanoplatform for colorectal cancer theranostics

Sihao Zhu, Ziyuan Li, Dongye Zheng, Yue Yu, Jing Xiang, Xiao Ma, Dongqing Xu, Jiajun Qiu, Ziyu Yang, Zhiyi Wang, Jun Li, Hongfang Sun, Weiqiang Chen, Xiangxi Meng, Yanye Lu, and Qiushi Ren

Table S1 Identification of optimal method to load Dox in PEGylated-PLGA nanoparticles

No.	PLGA-b-PEG (mg/mL)	Drug phase	Organic solvents	Surfactant for emulsification	Dox (mg)	Mean size[a] (nm)	PDI[a]	ζ potential[a]	Encapsulation efficiency (%)
1	20	Water	DCM	0.5% Span, 0.5% PVA	2	108.4	0.145	-15.6	27.46
2	20	Oil	DCM	0.5% Span, 0.5% PVA	2	120.3	0.173	-14.7	25.26
3	10	Water	DCM	0.5% Span, 0.5% PVA	2	53.7	0.196	-15.2	24.4
4	20	Oil	THF	0.5% PVA	2	245.4	0.282	-19.7	10.78
5	20	Water	DCM	—	2	545.6	0.396	-14.3	38.11
6	20	Water	DCM	0.5% span, 0.5% PVA	3	113.7	0.164	-13.2	29
**7**	**10**	**Water**	**DCM**	**0.5% span, 0.5% PVA**	**3**	**54.83**	**0.171**	**-13.8**	**54.51**
8	20	Oil	DCM	0.5% Span, 0.5% PVA	3	96.4	0.247	-12.9	30.77
9	20	Oil	THF	0.5% PVA	3	274.2	0.375	-15.6	40.52
10	20	Water	DCM	—	3	543.7	0.631	-19.3	58.74

[a] Note: average of three DLS measurements.

Table S2 Identification of optimal Method To Co-load miR-190-Cy7 and Dox in PPDCNPs.

No.	PLGA-b-PEG (mg/mL)	DC-chol (mg/mL)	Surfactant for emulsification	Drug concentration		Mean size (nm)[a]	PDI[a]	ζ potential[a]	Encapsulation efficiency (%)	
				Dox (mg)	miR-190-Cy7 (nmol)				Dox	miR-190-Cy7
1	10	0	0.5% Span 0.5% PVA	3	15	58.63	0.16	-23.23	49.60	19.34
2	10	0.1	0.5% Span 0.5% PVA	3	15	68.06	0.158	-7.75	47.22	80.65
3	**10**	**0.2**	**0.5% Span 0.5% PVA**	**3**	**15**	**90.43**	**0.173**	**2.45**	**46.01**	**88.33**
4	10	0.3	0.5% Span 0.5% PVA	3	15	103.76	0.193	7.64	21.88	90.39
5	10	0.5	0.5% Span 0.5% PVA	3	15	151.13	0.214	15.48	9.40	91.71
6	10	1.0	0.5% Span 0.5% PVA	3	15	193.56	0.232	27.14	2.50	93.22

[a] Note: average of three DLS measurements.

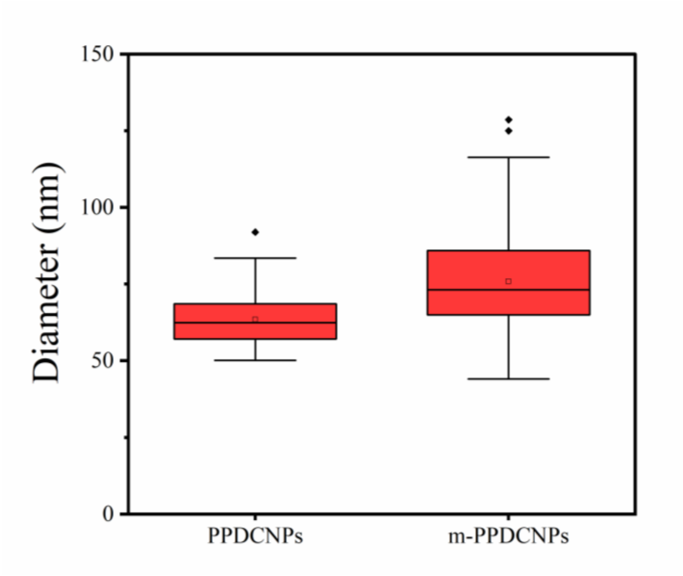

Figure S1 Size distribution of around 100 nanoparticles.

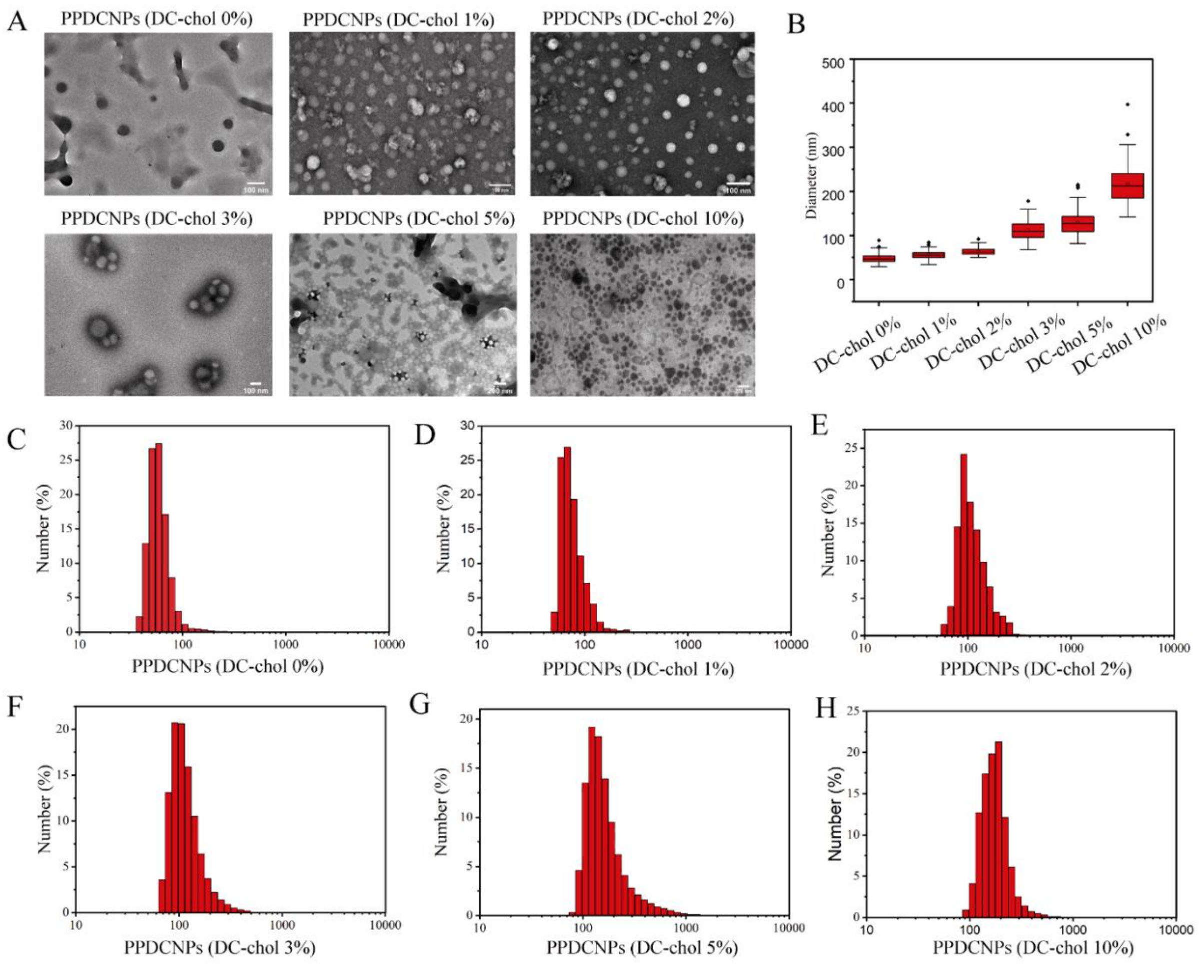

Figure S2 TEM images and hydrodynamic sizes of PPDCNPs.

(A) TEM image of PPDCNPs with different concentrations of DC-chol. (B) Size distribution of around 100 nanoparticles chosen from A. (C-F) Hydrodynamic size of miR-190 and Dox encapsulated PPDCNPs measured by dynamic light scattering.

Note: The figure on first row third column was also used in manuscript Figure 2D.

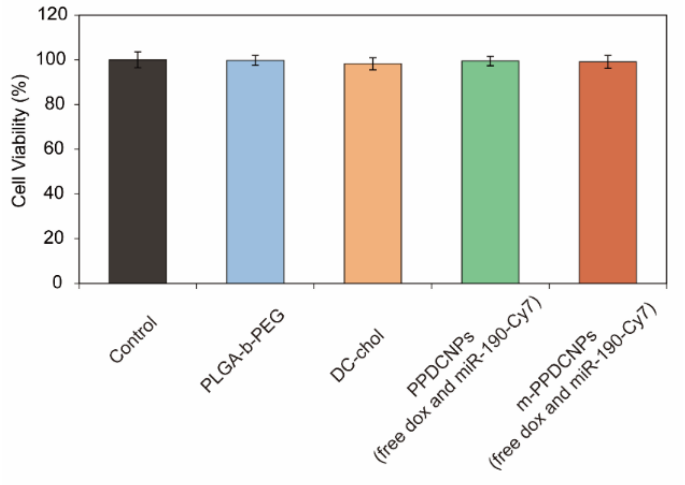

Figure S3 Cytotoxicity analysis of m-PPDCNPs.

Proliferation profile of HCT116 cells incubated with PBS, 10 mg/mL PLGA-b-PEG, 0.2mg/ml DC-chol, 10mg/ml PPDCNPs (free miR-190-Cy7 and Dox), 10 mg/mL m-PPDCNPs (free miR-190-Cy7 and Dox) for 24 h to analyze the Cytotoxicity of m-PPDCNPs.

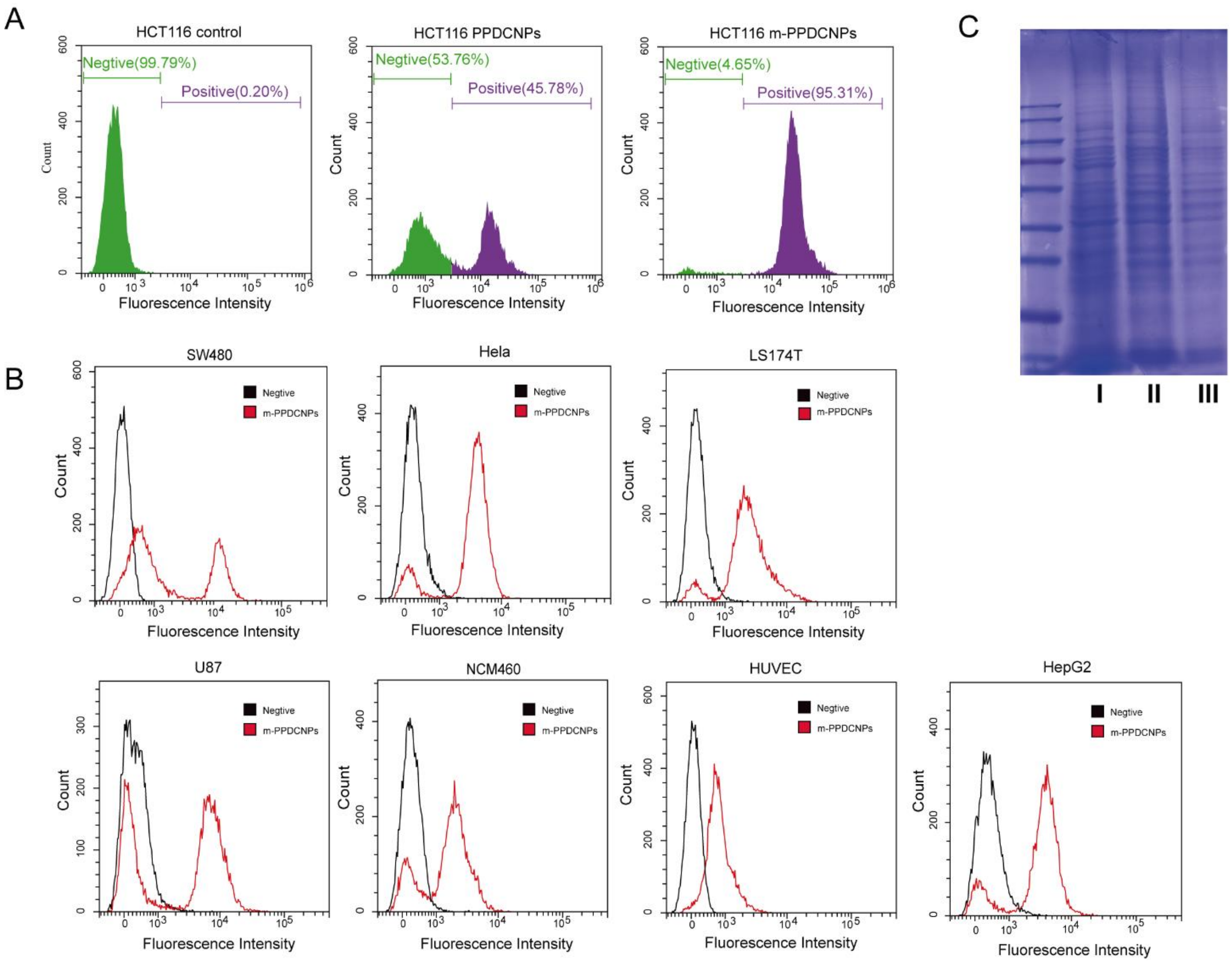

Figure S4 *In vitro* CRC-targeting ability of the miR-190-Cy7 and Dox loaded m-PPDCNPs.

(A) Flow cytometric profiles of HCT116 incubated with PPDCNPs and m-PPDCNPs. (B) Flow cytometric profiles of the seven cell lines SW480, LS174T, Hela, HepG2, U87, NCM460, and HUVEC upon 2 h incubation with m-PPDCNPs. (C) SDS-PAGE method to analyze protein composition. I: HCT116 Cancer cell total protein, II: cancer cell membrane protein, III: m-PPDCNPs.

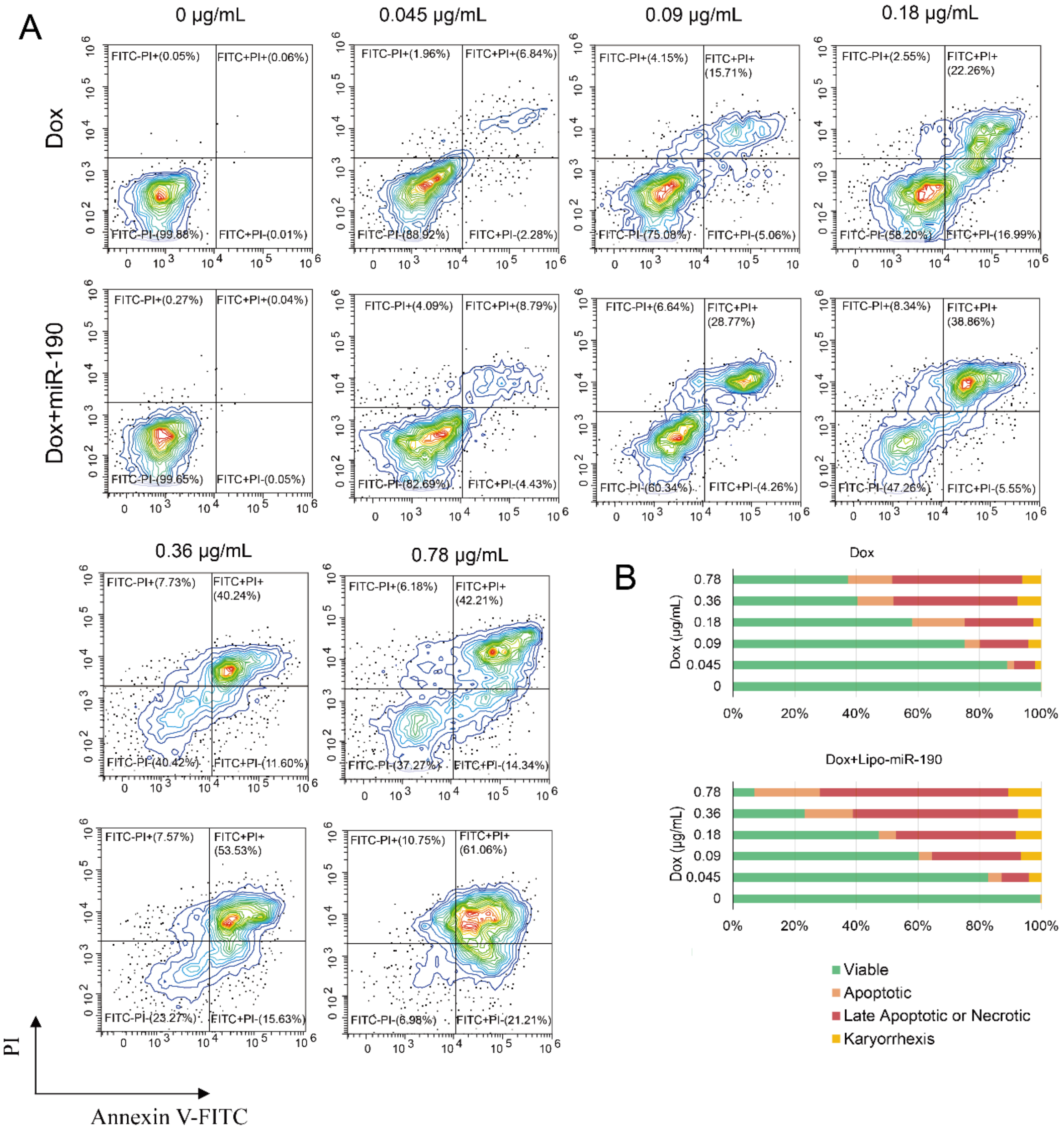

Figure S5 Flow cytometry analysis of apoptosis of HCT116 cells using Annexin V-FITC and PI staining.

(A) Apoptosis of HCT116 cells treated with Lipo3000-miR-190 and Dox with gradient concentration(0 μg/mL, 0.045 μg/mL, 0.09 μg/mL, 0.18 μg/mL, 0.36 μg/mL, 0.78 μg/mL) by flow cytometry analysis using Annexin V-FITC and PI staining, H_2O_2-treated cells were used as a positive control. (B) Apoptosis analysis of HCT116 cells by FACs.

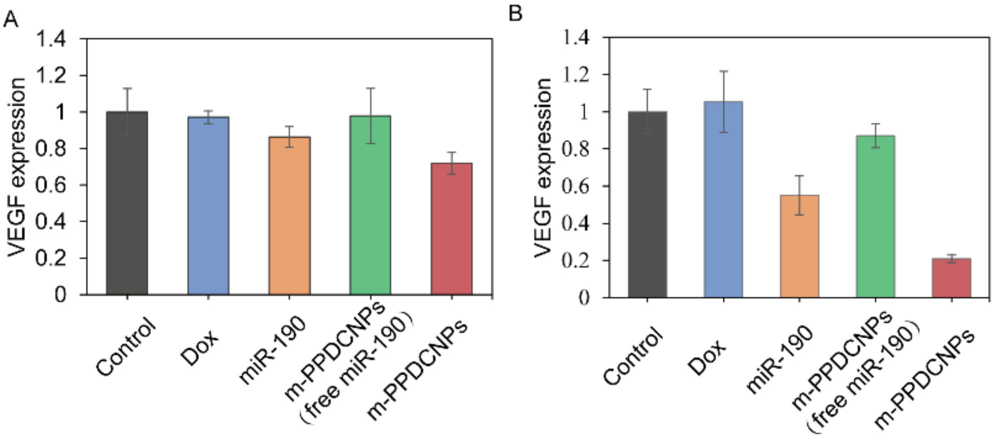

Figure S6 Western blot analysis and quantification of VEGF expression in the HCT116 tumor tissue after systemic treatment by PBS, miR-190, Dox, m-PPDCNPs (free miR-190-Cy7) and m-PPDCNPs.

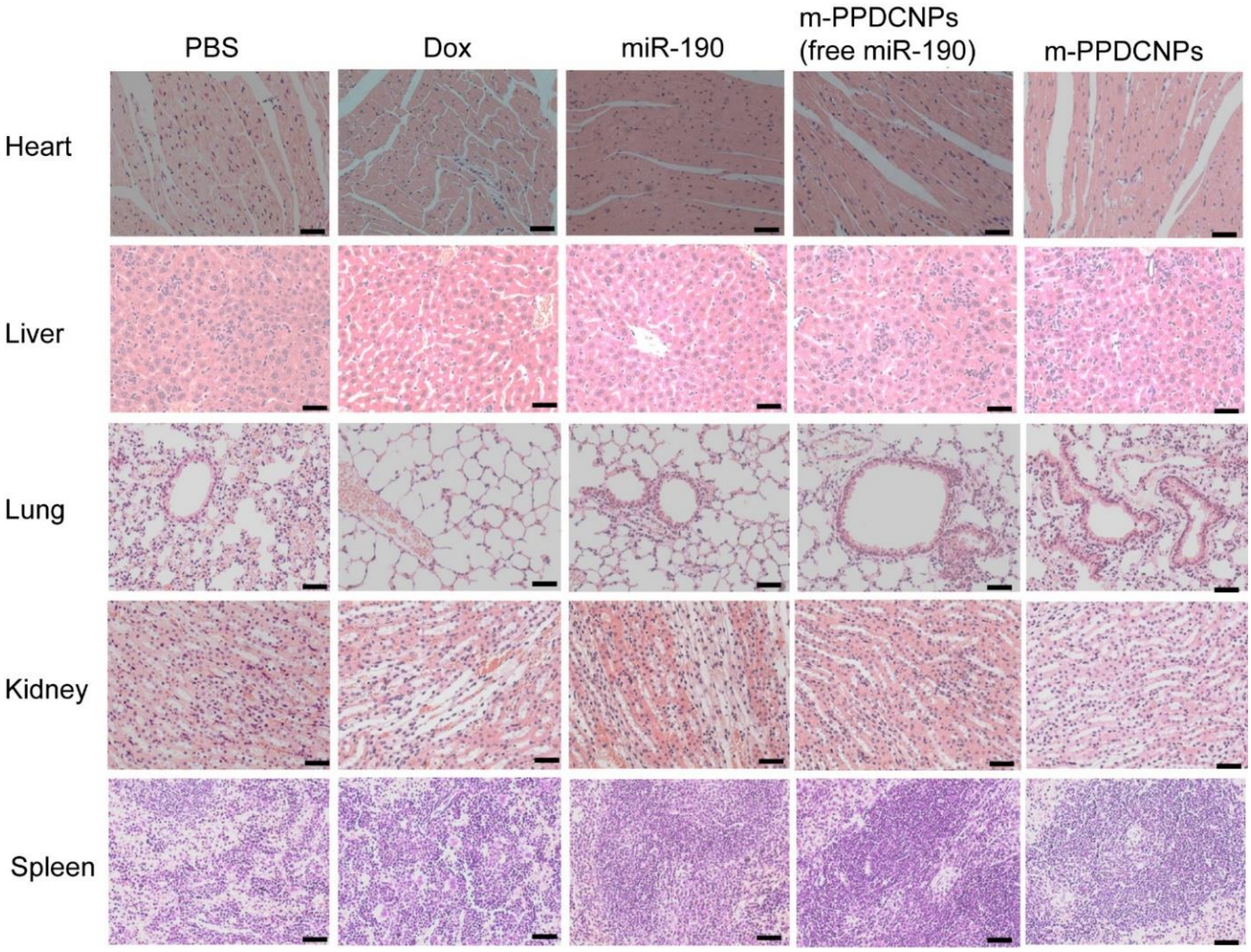

Figure S7 In vivo safety of m-PPDCNPs assessed by histopathological analysis.

Histological sections of the major organs (including heart, liver, lung, kidney, spleen) of mice intravenously injected with PBS, Dox, miR-190, m-PPDNPs (free miR-190) and m-PPDNPs for evaluating the safety and systemic toxicity in vivo of each group after 22 treatment. Hematoxylin-eosin; Scale bar: 50 μm.

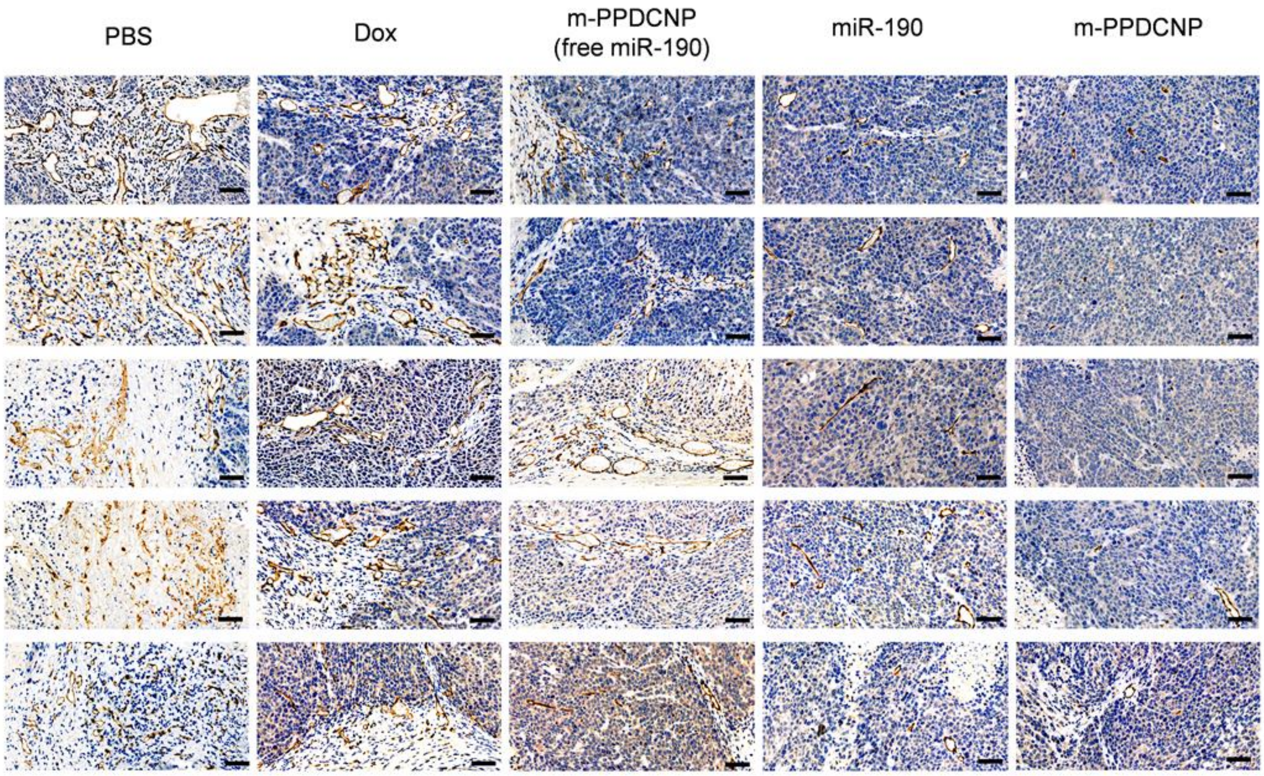

Figure S8 Immunohistochemistry CD31 staining sections of tumor.

Immunohistochemistry CD31 staining sections of primary tumors of HCT116 xenograft tumor-bearing nude mice after treatment by PBS, miR-190, and Dox and m-PPDCNPs (free miR-190-Cy7). Scale bar: 50 μm.

Note: The figures on first line were also used in manuscript Figure 6G.

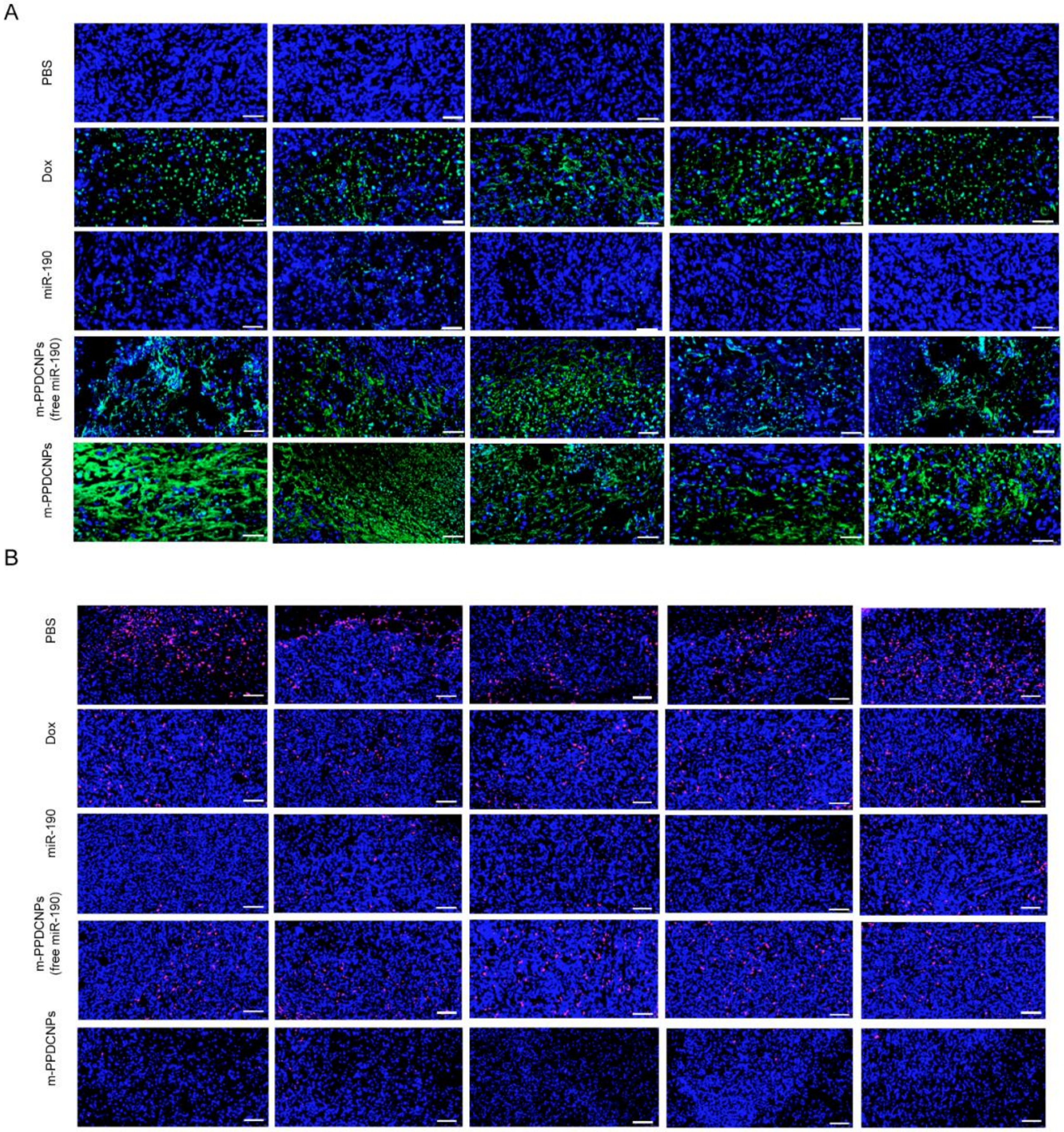

Figure S9 Immunofluorescent staining of TUNEL or KI67 expression in tumor.

Immunofluorescent staining of TUNEL(A) or KI67 (B) expression in tumor tissue sections of HCT116 xenograft tumor-bearing nude mice after treatment by PBS, miR-190, Dox and m-PPDCNPs (free miR-190-Cy7). Scale bar: 50 μm.

Note: The first column of (A) and (B) were also used in manuscript Figure 6H.
