# Supplementary material for: A cancer cell membrane coated, doxorubicin and microRNA co-encapsulated nanoplatform for colorectal cancer theranostics
Source: Mol Ther Oncolytics. 2022 Dec 7;28:182–96. doi: 10.1016/j.omto.2022.12.002 (PMC9937835; doi:10.1016/j.omto.2022.12.002)
Supplement: Document S2. Article plus supplemental information [file mmc2.pdf]

Molecular Therapy
Oncolytics
Original Article

A cancer cell membrane coated, doxorubicin and microRNA co-encapsulated nanoplatform for colorectal cancer theranostics

Sihao Zhu,[1,2,3,4,5,17] Ziyuan Li,[1,2,3,4,5,17] Dongye Zheng,[1,2,3,4,5] Yue Yu,[6] Jing Xiang,[1,2,3,4,5] Xiao Ma,[7] Dongqing Xu,[8] Jiajun Qiu,[9] Ziyu Yang,[10] Zhiyi Wang,[11] Jun Li,[12] Hongfang Sun,[1] Weiqiang Chen,[13,14] Xiangxi Meng,[15,16] Yanye Lu,[1,2,3,5] and Qiushi Ren[1,3,4,5]

[1]Department of Biomedical Engineering, College of Future Technology, Peking University, Beijing 100871, China; [2]Institute of Medical Technology, Peking University Health Science Center, Peking University, Beijing 100191, China; [3]National Biomedical Imaging Center, Peking University, Beijing 100871, China; [4]Institute of Biomedical Engineering, Shenzhen Bay Laboratory, Shenzhen 5181071, China; [5]Institute of Biomedical Engineering, Peking University Shenzhen Graduate School, Shenzhen 518055, China; [6]Beijing National Laboratory for Molecular Sciences (BNLMS), Key Laboratory of Bioorganic Chemistry and Molecular Engineering of Ministry of Education, College of Chemistry and Molecular Engineering, Peking University, Beijing 100871, China; [7]Research Group Signal Transduction, Department of Psychiatry, Ludwig Maximilian University of Munich, Nussbaumstr.7, 80336 Munich, Germany; [8]Department of Pediatric Hematology/Oncology, Xin Hua Hospital Affiliated to Shanghai Jiao Tong University School of Medicine, Shanghai 200092, China; [9]Department of Otolaryngology Head and Neck Surgery, the Ninth People's Hospital, Shanghai Jiao Tong University School of Medicine, Shanghai 200092, China; [10]College of Physics and Optoelectronic Engineering, Shenzhen University, Shenzhen 518060, China; [11]School of Chemistry and Chemical Engineering, South China University of Technology, Guangzhou 510640, China; [12]Laboratory Animal Center, Peking University, Beijing 100871, China; [13]Institute of Modern Physics, Chinese Academy of Sciences, Lanzhou 730000, Gansu Province, China; [14]Key Laboratory of Heavy Ion Radiation Biology and Medicine of Chinese Academy of Sciences, Key Laboratory of Basic Research on Heavy Ion Radiation Application in Medicine, Lanzhou 730000, Gansu Province, China; [15]Key Laboratory of Carcinogenesis and Translational Research (Ministry of Education/Beijing), Department of Nuclear Medicine, Peking University Cancer Hospital & Institute, Beijing 100142, China; [16]NMPA Key Laboratory for Research and Evaluation of Radiopharmaceuticals, Beijing 100142, China

Endogenous microRNAs (miRNA) in tumors are currently under exhaustive investigation as potential therapeutic agents for cancer treatment. Nevertheless, RNase degradation, inefficient and untargeted delivery, limited biological effect, and currently unclear side effects remain unsettled issues that frustrate clinical application. To address this, a versatile targeted delivery system for multiple therapeutic and diagnostic agents should be adapted for miRNA. In this study, we developed membrane-coated PLGA-b-PEG DC-chol nanoparticles (m-PPDCNPs) co-encapsulating doxorubicin (Dox) and miRNA-190-Cy7. Such a system showed low biotoxicity, high loading efficiency, and superior targeting ability. Systematic delivery of m-PPDCNPs in mouse models showed exceptionally specific tumor accumulation. Sustained release of miR-190 inhibited tumor angiogenesis, tumor growth, and migration by regulating a large group of angiogenic effectors. Moreover, m-PPDCNPs also enhanced the sensitivity of Dox by suppressing TGF-β signal in colorectal cancer cell lines and mouse models. Together, our results demonstrate a stimulating and promising m-PPDCNPs nanoplatform for colorectal cancer theranostics.

INTRODUCTION

Cancer development is triggered and actuated by intricate molecular interactions, whereby several families of non-coding RNAs play a subtle role. Among them, microRNAs (miRNA) are involved in various biological functions, modulating multiple aspects of neoplastic transformation and progression.[1–3] The pharmacotherapy based on miRNA was thus proposed and promoted to explore a new possibility in cancer management.[4–6] Therapeutics involving miRNA brings new opportunities to various types of tumors, but challenges still exist.

Colorectal cancer (CRC) is one of the major diseases threatening human health.[7] As concluded from clinical experience, a one-size-fits-all strategy fails to cope with the complexity of cancer. Surgical resection and chemotherapy are often the preferred strategies for CRC. Unfortunately, surgical resection comes with manifold contradictions, and the effect is significantly compromised by subsequent recurrence and metastasis.[8] In the meantime, cytotoxic compounds including doxorubicin (Dox), 5-fluorouracil, and capecitabine have been routinely used in the chemotherapy of CRC.[9,10] In practice, side effects due to off-target cytotoxicity induce patient intolerance, and the malignancy gradually

Received 16 February 2022; accepted 5 December 2022;
https://doi.org/10.1016/j.omto.2022.12.002.

[17]These authors contributed equally

Corresponding author.
E-mail: mengxiangxi@pku.edu.cn
Corresponding author.
E-mail: yanye.lu@pku.edu.cn
Corresponding author.
E-mail: renqsh@coe.pku.edu.cn

This is an open access article under the CC BY license (http://creativecommons.org/licenses/by/4.0/).

develops resistance. Given these issues, it is urgent to find comprehensive strategies to directly intervene in tumor biological behaviors.

Angiogenesis is a classic target for implementation of such strategies.[11,12] The formation of new blood vessels is a multi-step process which includes endothelial cell proliferation, migration, blood vessel formation, and cell survival. The umbilical role of the vascular endothelial growth factor (VEGF) signaling pathway in various cancer angiogenesis mechanisms has also become apparent.[13,14] Dozens of VEGF inhibitors have been approved or are being clinically evaluated for the treatment of various aggressive tumors. However, these VEGF inhibitors only achieve short-term improvement in many cases and are associated with drug resistance.[15–18] RNA technologies, especially miRNA, offer a uniquely therapeutic and diagnostic solution to these biological challenges. A member of miRNA named miR-190 has been revealed to greatly inhibit the expression of VEGF while interacting with multiple angiogenesis targets including TCF4, K-ras2, and Jak2. This miRNA also binds two diffusible growth factors, hepatocyte growth factor and insulin-like growth factor 1, thereby influencing the local microenvironment and subsequently regulating the expression of VEGF in adjacent endothelial cells. Moreover, miR-190 has been shown to restrain Smad2/4, which act as key transcription factors in the transforming growth factor β (TGF-β) signaling pathway in CRC.[19] The TGF-β signal promotes cell stemness and suppresses the sensitivity to anticancer drugs, and the effect is suspended with the inhibition of Smad2/4.[20,21] It is thus inferred that miR-190 may increase chemotherapy sensitivity and reduce drug resistance by inhibiting the TGF-β/Smad signaling pathway. All these properties suggest that miR-190 is promising against CRC.

However, challenges still exist in the *in vivo* delivery of miRNA, including rapid RNA degradation, easy recognition and clearance by the immune system, and off-target effects.[22] Emerging nanobiotechnology, cancer cell membrane (CCM)-coated nanoparticles, has been applied in various therapeutic and diagnostic practices, including but not limited to drug delivery.[23–28] Such a bionic nanotechnology uses CCMs with unique biological components, and has been effectively combined with different types of nanoparticles to improve targeted delivery.[29–31] The efficient enrichment of nanoparticles at the tumor site can be attributed to homologous targeting and immune escape. However, the co-delivery of multiple payloads including miRNA and chemotherapy drugs using CCMs remains largely unexplored.

Here, we report a nanoparticle platform—membrane-coated PLGA-b-PEG DC-chol nanoparticles (m-PPDCNPs)—that enables efficient co-delivery of miR-190-Cy7 and Dox with higher homologous targeting ability for drug/miRNA and better fluorescence imaging capability. Furthermore, we also demonstrate an integrated therapy and monitoring of CRC animal models based on our m-PPDCNPs platform. As shown in Figure 1, 3β-[N-(N,N-dimethylaminoethane)carbamoyl]cholesterol hydrochloride (DC-chol) was first incorporated into poly(lactide-co-glycolide)-b-poly(ethylene glycol)-b-poly(lactide-co-glycolide) (PLGA-b-PEG) to prepare PPDCNPs, which dramatically increased the miRNA loading efficiency while co-delivering Dox. Next, the cell membrane of colon cancer cell HCT116 was extracted, PEGylated with 1,2-distearoyl-*sn*-glycero-3-phospho-N-poly(ethylene glycol)ethanolamine (DSPE-PEG), and covered on the surface of the nanoparticles by extrusion, to form m-PPDCNPs. Combined with the homologous targeting and immune-escape characteristics of the CCM, the efficient enrichment of nanoparticles at the tumor site was realized. The sustained-release characteristics made the combined treatment of miR-190 and Dox feasible. From the start of treatment until 22 days after caudal intravenous therapy of m-PPDCNPs, tumor volume decreased by 75% and VEGF expression decreased by 80%. *In vivo* fluorescence imaging was performed to track the distribution of the targeted delivery system using the near-infrared dye Cyanine 7 (Cy7) conjugated to miR-190. Thus, this theranostic strategy offers a versatile platform for the intervention of CRC via miRNA and cytotoxic therapeutic agents.

RESULTS

Preparation and characterization of m-PPDCNPs

CCM-encapsulated nanoparticles (m-PPDCNPs) were manufactured via the extrusion method, using the extracted CCM and the miRNA/Dox-loaded polymeric nanoparticle PPDCNP. PPDCNP was obtained through a water-in-oil-in-water double emulsion method, involving the copolymer PLGA-b-PEG, amphiphilic molecule DC-chol, and the payload miR-190-Cy7 and Dox. The CCM was extracted from the HCT116 cell line, and DSPE-PEG was added to improve biocompatibility (Figure 1).

It has been reported that PLGA nanoparticles loaded with small interfering RNA and chemotherapeutic drugs were prepared by the method of complex emulsion-solvent evaporation.[32–34] The main issue is the inefficiency of miRNA encapsulation, which limits the application of PLGA-b-PEG as an miRNA delivery carrier.[35] To achieve high co-loading efficiency of miRNA and Dox, we optimized the concentrations of DC-chol and PLGA-b-PEG, the concentrations of miRNA and Dox, the amount of surfactant, and the solvent for the organic phase. Finally, we yielded a nanoparticle with optimal loading efficiency. To improve the loading efficiency of miRNA while ensuring that of Dox, we selected a better method by changing the preparation conditions. The hydrodynamic sizes and ζ-potential were measured for the product of each scheme (Table S1). The cationic molecule DC-chol determined the loading efficiency of miRNA and Dox by altering the ζ-potential and hydrophilicity, which in turn affected the size and the size distribution (Figures 2A, 2B, and S1).

The CCM-incorporated DSPE-PEG was used as "stealth" to enhance the uptake by homologous targeting ability, to extend long circulation time by the overexpressed immune-escape molecules such as CD47,[36–41] to reduce the non-specific binding between nanoparticles and serum proteins through PEG, and to avoid self-aggregation of related receptors on the cell membrane.[42] This would give the nanoparticles an invisible feature to avoid phagocytosis by phagocytes and removal of the reticuloendothelial system.[43,44] The obtained PPDCNP was subjected to CCM encapsulation via extrusion to obtain m-PPDCNPs. During this process, the size of the nanoparticle

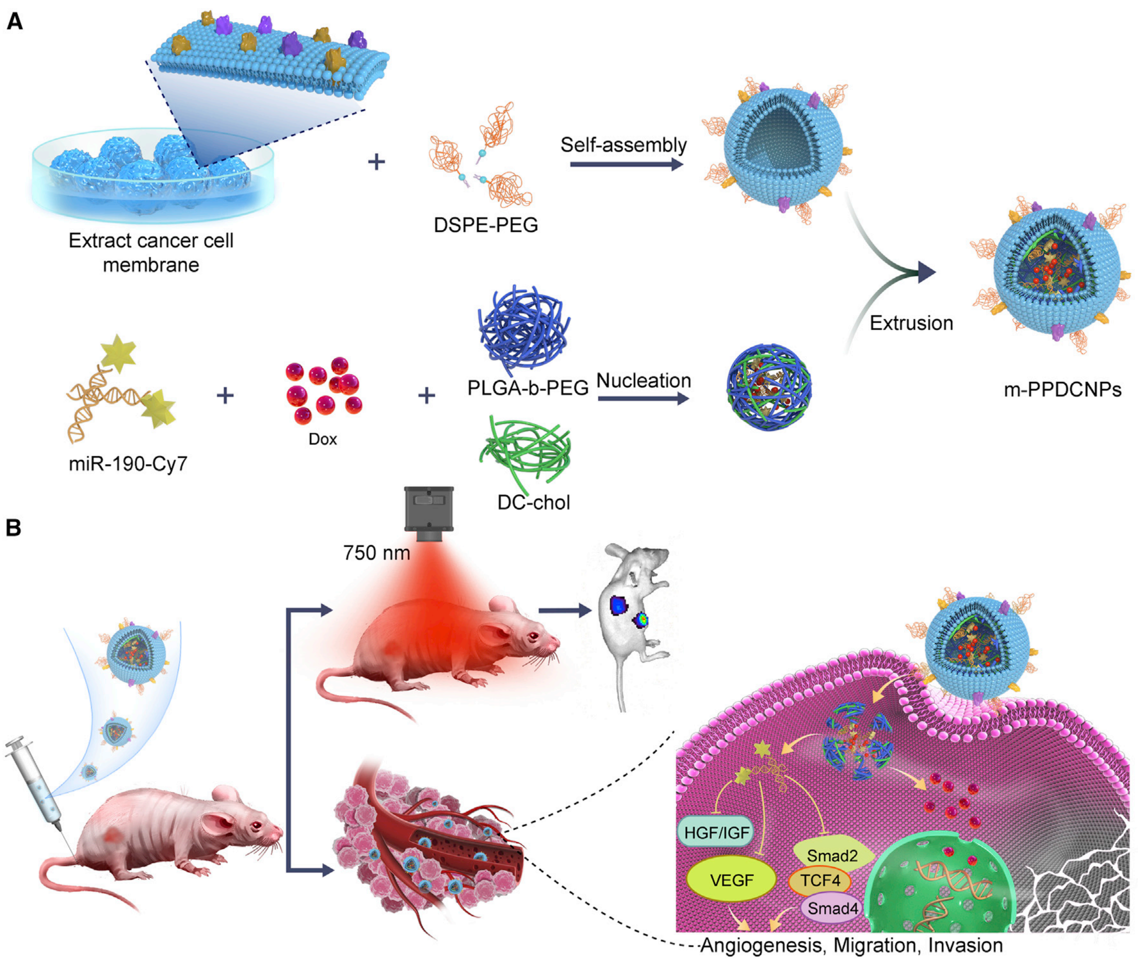

Figure 1. Illustration of the cancer cell membrane-biomimetic nanoparticles m-PPDCNPs encapsulating Dox and miR-190-Cy7 for targeting recognition of source cancer cell, microRNA delivery, fluorescence imaging, and combination therapy

(A) Preparation procedure of m-PPDCNPs. Extracted cancer HCT116 cell membrane, hybridized with PEGylated phospholipids (DSPE-PEG), was coated onto Dox and miR-190-Cy7 polymeric cores by extrusion. (B) Homologous-targeting m-PPDCNPs for in vivo microRNA delivery, fluorescence imaging, and combination therapy. After intravenous injection of m-PPDCNPs, through homologous targeting, m-PPDCNPs achieved perfect tumor aggregation, and realized tumor detection through Cy7. Release miR-190 through sustained-release to inhibit the expression of VEGF and inhibit tumor angiogenesis, combined with the Dox to inhibit tumor proliferation, and achieve integrated diagnosis and treatment.

was increased while the size distribution remained narrow, as demonstrated by the dynamic light-scattering measurements and transmission electron microscopy (TEM) (Figures 2C, 2D, and S2).

The miR-190-Cy7 and Dox release efficiency of PPDCNPs showed an initial burst release toward a sustained release within 6 days. Under 37°C and pH 6.0, PPDCNPs released miR-190-Cy7 with an amount of 63% at 48 h, then steadily increased to 81% on day 6 (Figure 2E). The release profile of Dox showed a similar trend under the same conditions, reaching a release amount of 63% (Figure 2F). This is consistent with relevant research reports.[45–47] The sustained release of the PPDCNPs delivery system could thus greatly reduce the side effects of anticancer drugs and alleviate the resistance of cancer cells to a large number of anticancer drugs.[48–50]

We screened a variety of feasible schemes to obtain the polymeric nanoparticles, and finally obtained a protocol that maximized the simultaneous loading of Dox and miR-190-Cy7. The key strategy is the incorporation of 2% DC-chol into the PLGA-b-PEG. With PPDCNPs, the encapsulation efficiency of Dox reached 46% while that of miR-190-Cy7 achieved 88%. This facile strategy effectively solves the inefficiency in miRNA loading of the PLGA-b-PEG system and provides an excellent nanodelivery platform for combination therapy. As a clinically approved agent, the PLGA polymer is generally considered safe, a major advantage over other biological materials with evident toxicity.[51–53] To the best of our knowledge, this system achieved the highest miRNA loading efficiency among all PLGA-based delivery systems to date. This facile manufacturing process ensures low polydispersity, stability, and non-toxicity of

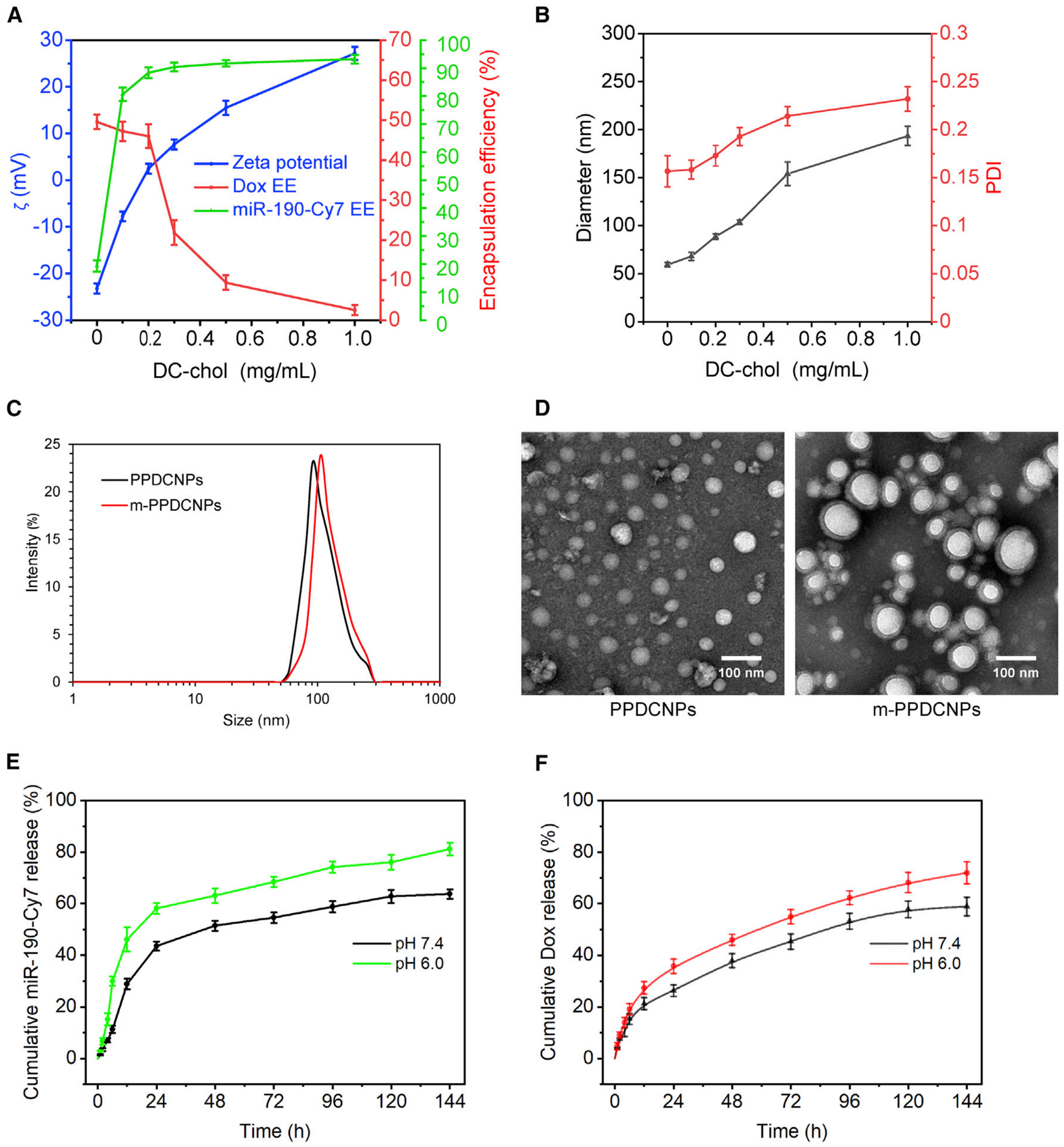

Figure 2. Characterization of m-PPDCNPs co-encapsulating Dox and miR-190-Cy7

(A) PPDCNPs and m-PPDCNPs sizes detected by dynamic light scattering (DLS). (B) TEM images of PPDCNPs and m-PPDCNPs. (C) Zeta potential (ζ) and encapsulation efficiency (EE%) of PPDCNPs with increasing concentration of DC-chol. (D) Size and polydispersity (PDI) of PPDCNPs with increasing concentration of DC-chol. (E and F) *In vitro* release of Dox and miR-190-Cy7 from PPDCNPs at pH of 6.0 and 7.4.

the nanoparticles obtained, implying an optimistic translational value.

Homologous targeting ability of m-PPDCNPs to colon cancer cell

Effective cell uptake is a prerequisite for gene silencing and combination medication.[54] The main obstacle to RNA delivery is the electronegativity of the glycoproteins and phospholipid bilayers on the surface of the cell membrane.[55,56] To prove that m-PPDCNPs were successfully functionalized by cancer cell adhesion molecules and escape molecules, we systematically studied the content of various proteins on the surface of m-PPDCNPs. Protein gel electrophoresis showed that m-PPDCNPs, CCMs, and HCT116 whole-cell protein had similar protein profiles (Figure S4C). Further western blot analysis was carried out to confirm the presence of specific homologous binding adhesion molecules on m-PPDCNPs. The results showed

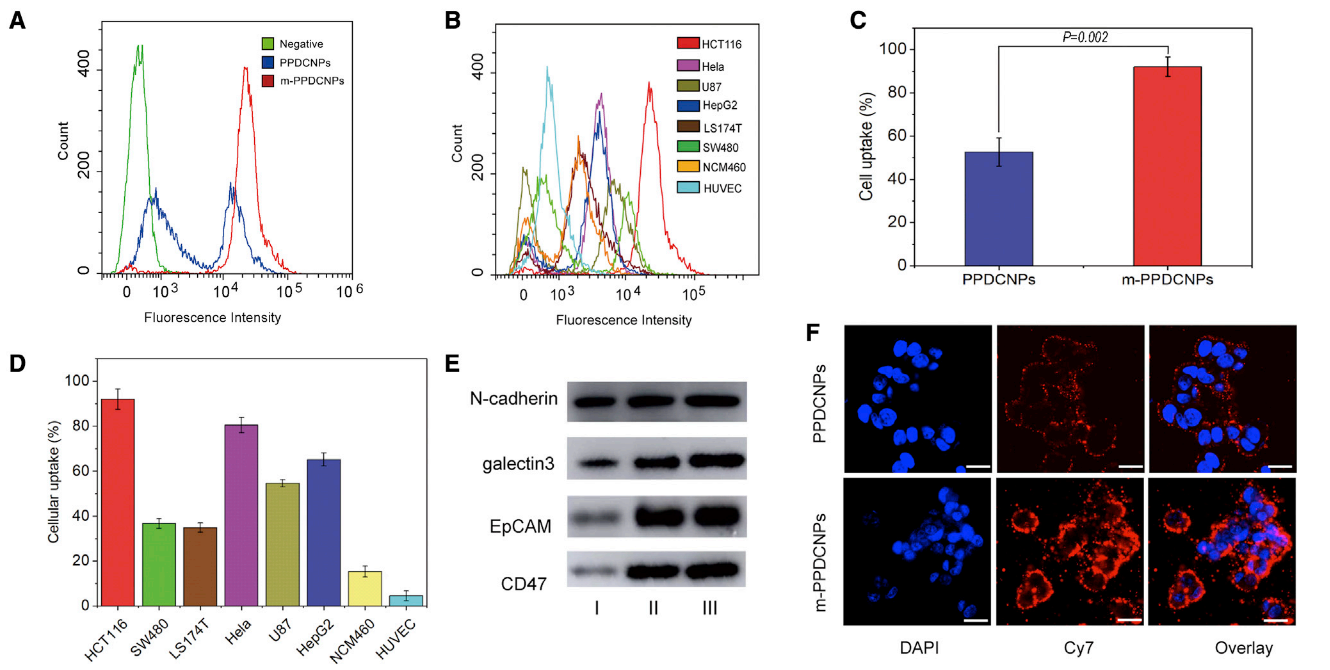

Figure 3. Homologous targeting ability of m-PPDCNPs to colon cancer cell

(A) Flow cytometric profiles of HCT116 incubated with PPDCNPs and m-PPDCNPs. (B) Flow cytometric profiles of the seven cell lines SW480, LS174T, Hela, HepG2, U87, NCM460, and HUVEC upon 2 h incubation with m-PPDCNPs. (C) The Cellular uptake ratio of HCT116 cells incubated with PPDCNPs and m-PPDCNPs. (D) The Cellular uptake ratio of eight cancer cells incubated with m-PPDCNPs. (E) Western blot analysis of the expression of membrane adhesion proteins (N-cadherin, galectin3 and EpCAM) and escape molecules (CD47). I: HCT116 Cancer cell total protein, II: cancer cell membrane protein, III: m-PPDCNPs. (F) Confocal microscopy images of HCT116 cells incubated with PPDCNPs and m-PPDCNPs. Nuclei were stained by DAPI (blue signal), miR-190- Cy7 was used (red signal). Scale bars, 25 μm.

that m-PPDCNPs also possessed these cell adhesion molecules (EpCAM, N-cadherin, and galectin-3). The particles specifically targeted cancer cells, confirming the specific recognition and binding ability between m-PPDCNPs and cancer cells. At the same time, we tested the immune-escape molecule CD47. With the same concentration of protein, the CCM endowed PPDCNPs with long blood circulation time and immune-escape characteristics (Figures 3E and S4D).

To verify that m-PPDCNPs had an outstanding ability to target the same type of cancer cells, we incubated PPDCNPs and m-PPDCNPs with colon cancer cell HCT116 for 2 h and used flow cytometry to detect the uptake capability. It was found that the uptake of PPDCNPs by HCT116 cells was 45%, and the uptake of m-PPDCNPs reached 95% (Figures 3A, 3C, and S4A). Next, we incubated m-PPDCNPs with seven other cell lines for 2 h and tested the cell uptake of HCT116 CRC membrane-encapsulating PPDCNPs (Figures 3B, 3D, and S4B). The results showed that the uptake of m-PPDCNPs by HCT116 was far stronger than that of other cancer cells, and the uptake by other cancer cells also exceeded that of normal tissue cells.

Meanwhile, we used the near-infrared confocal fluorescence microscope to observe the uptake of m-PPDCNPs by the HCT116 cell line. After 2 h of incubation, compared with PPDCNPs, m-PPDCNPs exhibited higher Cy7 fluorescence intensity in the cytoplasm of HCT116. This further proved that the nanoparticles wrapped in CCM could be absorbed by a homologous type of cancer cells far stronger than those not wrapped by the same membranes.[57–59] The results indicated that the m-PPDCNPs coated with CCM were subject to homologous targeting. The homologous targeting effect was outstanding, confirming the specific binding ability of m-PPDCNPs with HCT116 cells.

Verification of the effect of m-PPDCNPs *in vitro* gene silencing and combination medication

We next needed to determine whether m-PPDCNPs affect gene silencing efficiency. First, qRT-PCR determined that miR-190-Cy7 encapsulated in nanoparticles had a satisfactory result in inhibiting VEGF mRNA expression. We coated miR-negative control (miR-NC) and miR-190-Cy7 in nanoparticles to prepare PPDCNPs (only miR-NC) and PPDCNPs (only miR-190-Cy7). After incubating the PPDCNPs with the HCT116 cell line for 24 h, the VEGF mRNA expression of HCT116 cells was only 53% of the control group.

In cells treated with Lipofectamine 3000 (Lipo3000) delivering miR-190-Cy7, the expression of VEGF mRNA was 64% of the control group. After 48 h of incubation, the expression of VEGF mRNA in the PPDCNPs-treated group was only 21% of that in the control group,

while the Lipo3000 transfected group had a 47% VEGF mRNA expression. The results showed that PPDCNPs inhibited VEGF mRNA more effectively than Lipo3000. The inhibitory effect was sustained at 48 h, which reflected the sustained release of PPDCNPs and the ability to inhibit the expression of VEGF. The ability of m-PPDCNPs to inhibit VEGF mRNA was stronger than that of PPDCNPs. After 24 h of incubation of m-PPDCNPs, the expression of VEGF mRNA was only 39% of the control group. After 48 h, the ratio dropped to 11%. This also showed that with the same concentration of nanoparticles, homologous targeting has a greater advantage (Figures 4A and 4B).

Next, the Firefly luciferase reporter assay was used to validate whether m-PPDCNPs could inhibit the expression of VEGF. We first tested the PPDCNPs, which is only the miRNA (miR-190-Cy7) part of m-PPDCNPs. Co-transfecting VEGF 3′ UTR plasmid and PPDCNPs into HCT116 cells significantly decreased luciferase activity compared with the control (Figure 4C). The luciferase activity was reduced to 45% 24 h after co-transfection and continuously dropped to 16% in 48 h, suggesting that the expression of VEGF was negatively regulated by PPDCNPs at the messenger RNA level. Moreover, the inhibition ability was even more significant on m-PPDCNPs. The luciferase activity dropped to 30% in 24 h and 9% in 48 h, respectively, after co-transfection of VEGF 3′ UTR plasmid and m-PPDCNPs (Figure 4D). More importantly, none of the nanoparticles (free miR-190 and Dox) used in the experiment showed significant toxicity (Figure S3).

In tumor tissues, cancer cells promote angiogenesis by secreting large amounts of VEGF.[60–63] An ELISA experiment was used to detect the secretion of VEGF in HCT116 cells after incubation with m-PPDCNPs and PPDCNPs for 24 h and 48 h. The results showed that after 24 h of co-incubation, the amount of VEGF secreted by HCT116 cells in the PPDCNPs group decreased by 49%, and after 48 h the amount of VEGF secreted by the cells decreased to 13%. The amount of VEGF secreted by cancer cells in the m-PPDCNPs group was 35% and 2%, respectively. These results all showed that besides VEGF mRNA, PPDCNPs could also reduce VEGF protein both produced in the cell and secreted outside the cell.

The inhibitory function of suppressing expression was enhanced by the increased cellular uptake of the homologous targeting m-PPDCNPs (Figures 4E and 4F). Western blot was used to detect the expression of VEGF in HCT116 after incubating for 24 h and 48 h with m-PPDCNPs. The results showed that after 24 h of incubation, the amount of VEGF protein produced by HCT116 in the PPDCNPs group decreased by 82% and after 48 h, the amount of VEGF protein produced by the cells decreased to 33%. In the m-PPDCNPs group, the amount of VEGF protein expressed by cancer cells was 72% and 20%, respectively (Figures 4G and 4H). The results of the western blot analysis were similar to the results of VEGF mRNA detection, and both showed that m-PPDCNPs had the characteristics of inhibiting gene expression. This inhibitory effect could be continuously reduced for a long time through a sustained-release system.

The combination of multiple therapeutic agents can immediately target different cellular pathways, preventing the transformation of cancer cells into drug-resistant cells and inhibiting the survival of drug-resistant cell populations. Nanoparticles have been widely evaluated as a combination drug-delivery carrier for multiple drugs. CCK8 analysis was used to further investigate the proliferation effect of m-PPDCNPs on HCT116 cells. The results showed that m-PPDCNPs exhibited high proliferation inhibition ability compared with PPDCNPs, Lipo-(miR-NC), Lipo-miR-190-Cy7, PPDCNPs (Dox and miR-NC), and PPDCNPs. The effect was more obvious at later time points. The cell amount at 48 h was only 3% in the control group, indicating that miR-190 increased the cytotoxicity of Dox and had a continuous killing effect resulting from the sustained-release ability (Figure 4J). Thus, we verified that miR-190 could promote the sensitivity of HCT116 cells to Dox through CCK8 analysis (Figure 4I). The apoptosis under combination miR-190 and Dox was also quantitatively determined by fluorescence-activated cell sorting of staining cells with annexin V-fluorescein isothiocyanate and propidium iodide. Concordant with the CCK8 experiment, the apoptosis rate of cancer cells increased gradually with the increase of chemotherapy drug concentration. After addition of miR-190, the effect of this combination drug was enhanced, that is, miR-190 promoted the apoptotic effect of chemotherapy drugs on HCT116 colon cancer cells (Figure S5).

In vivo biodistribution of homologous targeting m-PPDCNPs

To evaluate the ability of CCM-encapsulated m-PPDCNPs to target tumors *in vivo*, we used an *In vivo* imaging system (IVIS) to study the biodistribution of m-PPDCNPs in nude mice carrying HCT116 xenograft tumors after tail vein injection (Figure 5A).

Figure 5A shows that free miR-190-Cy7 was quickly cleared from the body, and no obvious fluorescence signal was detected after 12 h. Compared with free miR-190-Cy7, PPDCNPs enhanced *in vivo* retention, and nanoparticles were observed to accumulate m-PPDCNPs in the tumor. With m-PPDCNPs, the tumor exhibited significant retention while the background signal from other organs faded at 24 h, outlining the superb tumor-targeting ability of the nanoparticles. To analyze the biodistribution of nanoparticles in major organs, we collected tumors and major organs of mice 24 h after injection from mice administered miR-190-Cy7, PPDCNPs, and m-PPDCNPs (Figures 5B and 5C). The amount of m-PPDCNPs in the spleen and kidneys was much lower than that in PPDCNPs, with decreases of 81% and 29%, respectively. The signal intensity in the liver and lung was also lower than that in the PPDCNPs group, with a decrease of 14% and 12%, respectively. Compared with PPDCNPs and free miR-190-Cy7, the tumor accumulation of m-PPDCNPs increased by 2.7-fold and 15.4-fold, respectively. Compared with the m-PPDCNPs group, the PPDCNPs lacking CCM coating had a much smaller number of nanoparticles accumulated in the tumor. This fully reflected the homologous targeting ability of CCM. These advantages can be of great significance in enhancing the delivery efficiency of miR-190-Cy7 and Dox.

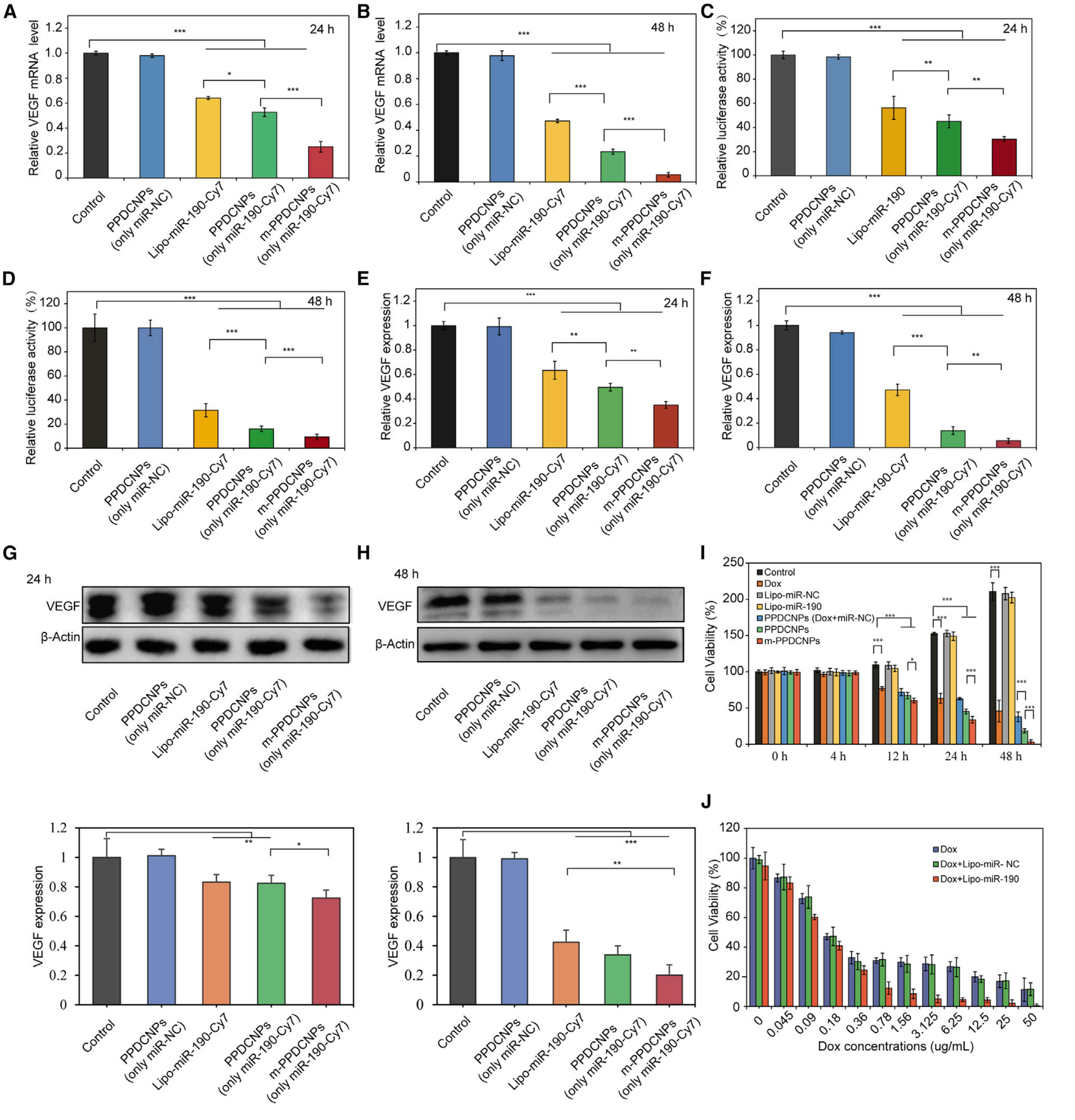

Figure 4. Verification of the effect of m-PPDCNPs *in vitro* gene silencing and combination medication

(A and B) Relative VEGF mRNA levels in HCT116 cells incubated with PPDCNPs (only miR-NC), Lipo-miR-190-Cy7 , PPDCNPs (only miR-190-Cy7) or m-PPDCNPs (only miR-190-Cy7) for 24 h or 48 h as indicated by real-time PCR. β-Actin was used as the loading control in real-time PCR assay. N = 3. * $P < 0.05$, ** $P < 0.01$, *** $P < 0.001$ (t-test). (C and D) Luciferase activity in the Luci-HCT116 cells upon transfection with PPDCNPs (only miR-NC), Lipo-miR-190-Cy7, PPDCNPs (only miR-190-Cy7) or m-PPDCNPs (only miR-190-Cy7) for 24 h or 48h. N = 3. ** $P < 0.01$, *** $P < 0.005$ (t-test). Control: Cells incubated with free medium. (E and F) ELISA assay of secreted VEGF in HCT116 cells incubated with PPDCNPs (only miR-NC), Lipo-miR-190-Cy7, PPDCNPs (only miR-190-Cy7) or m-PPDCNPs (only miR-190-Cy7) for 24 h or 48 h, as indicated. N = 3. * $P < 0.05$, ** $P < 0.01$, *** $P < 0.001$ (t-test). (G andH) Western blot analysis and quantification of VEGF expression in HCT116 cells treated with PPDCNPs (only miR-NC), Lipo-miR-190-Cy7, PPDCNPs (only miR-190-Cy7) or m-PPDCNPs (only miR-190-Cy7) for 24 h or 48 h. (I) Proliferation profile of HCT116 cells treated with Lipo-miR-190-Cy7 and Dox with gradient concentration. N = 5. (J) Proliferation profile of HCT116 cells incubated with Dox, Lipo-miR-NC, Lipo-miR-190-Cy7, PPDCNPs (only miR-NC), Lipo-miR-190-Cy7, PPDCNPs (Dox and miR-NC), PPDCNPs or m-PPDCNPs for 0 h, 4 h, 12 h, 24 h or 48 h, as indicated. N=5.

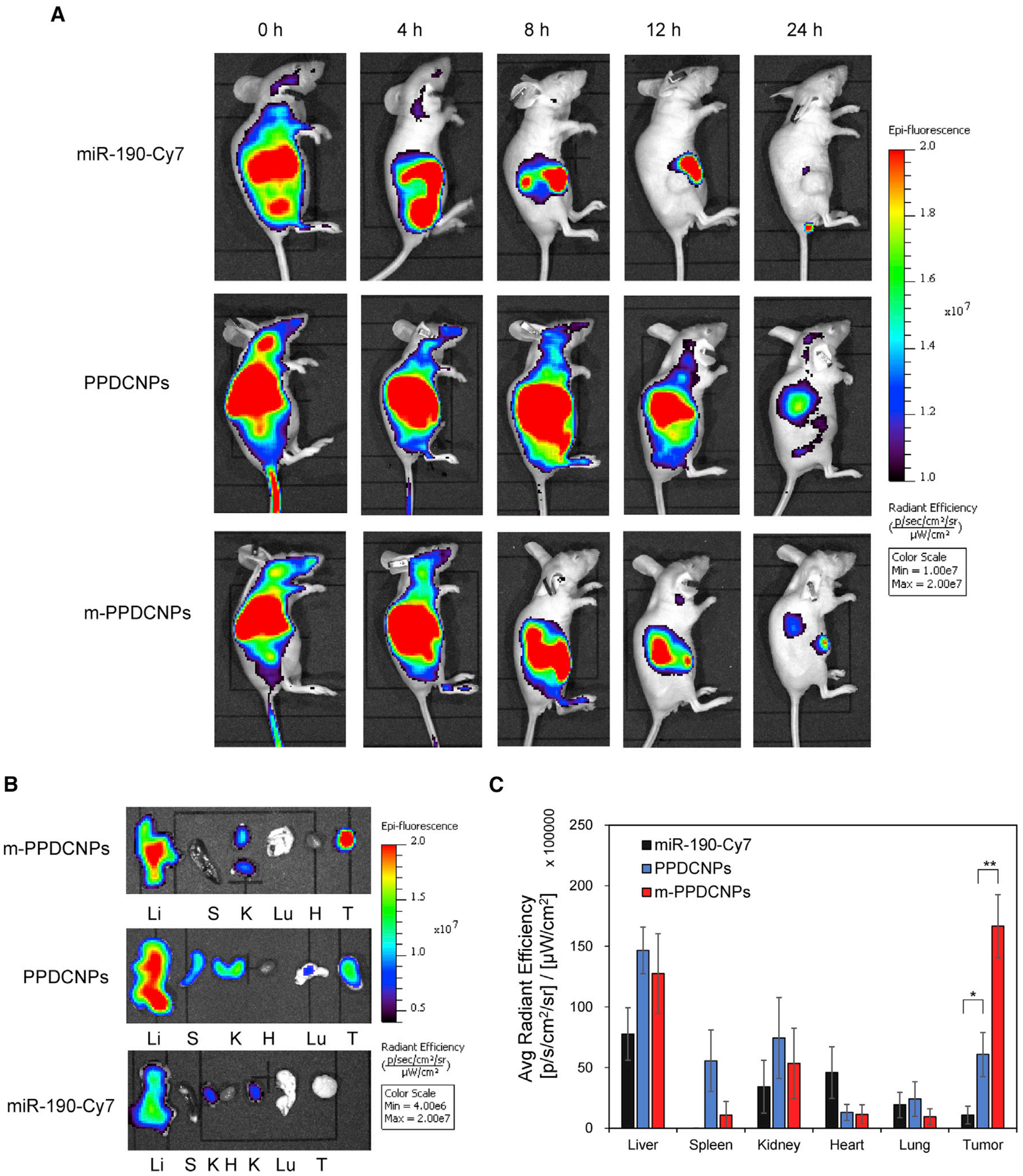

Figure 5. *In vivo* biodistribution of homologous targeting m-PPDCNPs

(A) Overlaid Cy7 fluorescent image of the HCT116 xenograft tumor-bearing nude mice 0 h, 4 h, 8 h, 12 h and 24 h post-injection of naked miR-190-Cy7, PPDCNPs, and m-PPDCNPs. (B) Overlaid Cy7 fluorescent image of the tumors and main organs of the HCT116 xenograft tumor-bearing nude mice sacrificed 24 h post-injection of naked miR-190-Cy7, PPDCNPs, and m-PPDCNPs. Li: liver, S: Spleen, K: Kidney, H: Heart, Lu: lung, T: tumor. (C) Biodistribution of the naked miR-190-Cy7, PPDCNPs, and m-PPDCNPs quantified from (B).

In vivo gene silencing and antitumor research of combination medication and antitumor angiogenesis

To evaluate the antitumor ability of m-PPDCNPs camouflaged by CCM, we prepared a nude mouse model of HCT116 colon cancer xenograft tumor. After the tumor volume reached 100 mm^3, the nude mice were randomly divided into five groups, and the tumor-carrying nude mice in each group were injected with PBS, miR-190-Cy7 (20 nmol/kg body weight), Dox (2 mg/kg body

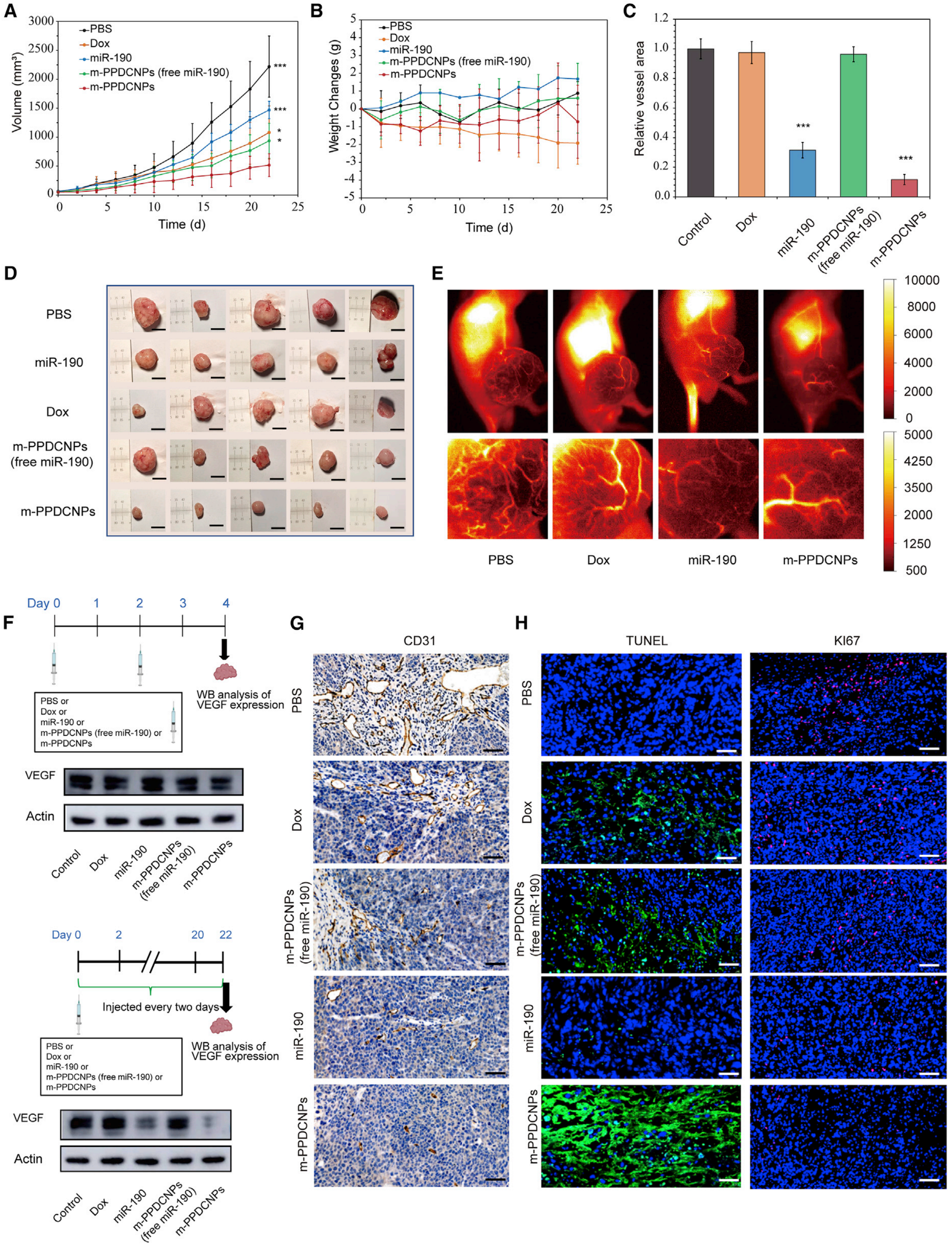

(legend on next page)

weight), and CCM-coated m-PPDCNPs (free miR-190-Cy7) and m-PPDCNPs (25 mg/kg), respectively. The treatment lasted continuously for 22 days, and the injection was performed once every 2 days. As shown in Figure 6A, tumor growth in the m-PPDCNPs group was significantly inhibited. The m-PPDCNPs (free miR-190) in the Dox group also inhibited the rapid proliferation of tumors, but the therapeutic effect was significantly weaker than that of the m-PPDCNPs. This showed that Dox combined with miR-190 could achieve a superior therapeutic effect through the targeted delivery system. After the treatment, the tumors were dissected from the tumor-carrying nude mice. The tumor size of the m-PPDCNPs group were visually compared with those of the other control groups and demonstrated a clear and good therapeutic effect (Figure 6D). After treatment with m-PPDCNPs, there was no significant change in the weight of the mice. However, in the Dox group the weight of the mice decreased rapidly (Figure 6B). The toxicity and side effects of Dox treatment might be related to this phenomenon. Extensive studies have found that Dox has strong cardiotoxicity and multi-organ-damaging characteristics,[64] and drug resistance is very common in Dox treatment, which is closely related to the TGF-β/Smad signaling pathway.[65,66] Hematoxylin and eosin (H&E) staining was used to evaluate *in vivo* safety and systemic toxicity of each group in the treatment regime (Figure S7). The hearts of mice treated with Dox showed severe damage and lymphocyte infiltration. Major organs (including heart, liver, spleen, lung, and kidney) of mice treated with Dox showed severe damage and lymphocyte infiltration. However, the toxicity of the internal organs was substantially weakened when using nanoparticle therapy. The treatment group with the targeted nanoplatform m-PPDCNPs did not show any significant organ pathology. Together, these results indicated the safety of m-PPDCNPs as a nano-co-delivery platform.

In addition, we aimed to evaluate the drug effect of tumor blood vessels after treatment in real time. Indocyanine green (ICG) NIR-II fluorescence imaging was used to evaluate the tiny blood vessels in the tumor area of tumor-carrying mice. The second near-infrared window (NIR-II) fluorescence imaging system was used to image the tumor blood vessels of tumor-carrying nude mice after injection of ICG through the tail vein. After 22 days of treatment, compared with other treatments the tumor blood vessels of tumor-carrying nude mice treated with m-PPDCNPs showed little sign of overgrowth and appeared to be normal, indicating that m-PPDCNPs can inhibit tumor angiogenesis effectively (Figure 6E). We used the antibody of the blood vessel marker CD31 to perform an immunohistochemistry (IHC) analysis of the tumor blood vessel. After verifying the results of fluorescence imaging, the vessel area of tumor by CD31 IHC staining treated with m-PPDCNPs was markedly reduced by about 86% (Figures 6C, 6G, and S8).

We next investigated the expression of VEGF in tumor tissues after treatment with m-PPDCNPs by western blotting. First, we evaluated the expression of VEGF inhibition effect of m-PPDCNPs. During the first 4 days of treatment, the expression of VEGF was significantly decreased by 29%. We then evaluated the inhibited expression of VEGF at the end of m-PPDCNPs treatment, whereby the expression of VEGF was dramatically reduced by 80% compared with the control group (Figures 6F and S6). Particularly worth mentioning is that miR-190 used in the experiment was modified in a variety of ways (including glycation, and cholesterol modification), and the direct injection of miR-190 through the tail vein also had an inhibitory effect on VEGF expression. This miRNA was used in the experiment to make the *in vivo* treatment more stable. To diagnose the microvasculature in tumor-bearing mice, improved ICG fluorescence angiography was conducted with an NIR-II IVIS to image the tumor vessels of tumor-bearing nude mice. This *in vivo* evaluation method revealed the effect of the combined treatment of miR-190 and Dox on superficial and some deeper tumor blood vessels, which reflected the effect of VEGF depletion. Combined with the CD31 immunohistochemical assay, it could be concluded that m-PPDCNPs had a good inhibitory effect on tumor blood vessels. The combined delivery strategy proposed enriches the current family of cancer biological therapies and could be further explored with different combinations of miRNA and drugs.

Finally, we evaluated the apoptosis and proliferation of tumor tissues after 22 days of treatment by immunofluorescence. Terminal deoxynucleotidyl transferase-mediated nick end labeling (TUNEL)—a relatively sensitive apoptosis detection method—was used to evaluate the therapeutic effect of m-PPDCNPs. The results showed that there was a large area of apoptotic signal in the tumors treated with m-PPDCNPs that was significantly stronger than the apoptotic signal in the m-PPDCNPs treatment group without miR-190. This also helps to demonstrate the effectiveness of combination drugs. Ki67 was used to evaluate the proliferation ability of tumors after receiving m-PPDCNPs, whereby the results showed only weak proliferation compared with the control group (Figures 6H and S9). In summary, the combined treatment of colon cancer with sustained-release m-PPDCNPs co-carrying Dox and miR-190 is a feasible and effective treatment method.

Figure 6. Antitumor effect and *in vivo* gene silencing of the m-PPDCNPs

(A) Tumor volume of the HCT116 xenograft tumor-bearing nude mice (N = 5) after treatment by PBS, miR-190, Dox, m-PPDCNPs (free miR-190-Cy7) and m-PPDCNPs. * $P < 0.05$; *** $P < 0.005$. (B) Weight changes of the HCT116 xenograft tumor-bearing nude mice after treatment by PBS, miR-190, Dox, m-PPDCNPs (free miR-190-Cy7) and m-PPDCNPs. (C, G) CD31 immunohistochemistry staining sections of primary tumors of HCT116 xenograft tumor-bearing nude mice after treatment by PBS, miR-190, Dox, m-PPDCNPs (free miR-190-Cy7) and m-PPDCNPs. *** $P < 0.005$. Scale bar, 50 μm. (D) Representative photos of mice bearing HCT116 tumors and excised tumors 22 d after treatments. (E) The ICG NIR-II fluorescence imaging evaluate the blood vessels in the tumor area of tumor-carrying mice. (F) Western blot analysis of VEGF expression in the HCT116 tumor tissue after systemic treatment by PBS, miR-190, Dox, m-PPDCNPs (free miR-190-Cy7) and m-PPDCNPs. (H) Immunofluorescent staining of TUNEL or Ki67 expression in tumor tissue sections of HCT116 xenograft tumor-bearing nude mice after treatment by PBS, miR-190, Dox, m-PPDCNPs (free miR-190-Cy7) and m-PPDCNPs. Scale bar: 50 μm.

DISCUSSION

The invention of co-encapsulated nanoparticles for cancer therapy is compelled to consummate several standards, i.e., containing non-toxic side effects, preventing miRNA from degradation by RNase, high loading efficiency, non-biotoxicity, outstanding targeting, avoiding wipeout by mononuclear macrophages, preferably with combination drug therapy, and precision guidance by imaging probes on the tumor. To address these issues, the m-PPDCNP was designed. This nanocomposite has several unique features: (1) the combined therapeutics featuring both miR-190 and Dox, enabling synergistic therapeutics; (2) exceptionally specific accumulation in tumors due to the combined effect of homologous targeting and immune escape; and (3) highly efficient loading, precise delivery, and sustained release of the encapsulated miRNA and drug, maintaining the pharmaceutical activity. With these traits, m-PPDCNPs demonstrated superior theranostic ability with the CRC animal model.

It has been proposed that, with its anti-angiogenesis potential, miR-190 might be a therapeutic candidate in cancer intervention, and, in combination with Dox, the effect could be amplified. In this study, the effect of m-PPDCNPs was explored and achieved a good therapeutic effect via precise delivery and sustained release of miR-190 and Dox. The tumor angiogenesis was inhibited by miR-190, which inhibited the production of key factor VEGF, as demonstrated in molecular, transcriptional, protein-related, and histological scopes. Meanwhile, miR-190 promoted the sensitivity of colon cancer cell HCT116 to Dox by inhibiting the TGF-β signaling pathway, which significantly enhanced the apoptotic effect of the cells and realized the combination therapy. To diagnose the microvasculature in tumor-bearing mice, improved ICG fluorescence angiography was conducted with an NIR-II IVIS to image the tumor vessels of tumor-bearing nude mice. This *in vivo* evaluation method revealed the effect of the combined treatment of miR-190 and Dox on superficial and some deeper tumor blood vessels, which reflected the effect of VEGF depletion. Combined with the CD31 immunohistochemical assay, it could be concluded that m-PPDCNPs had a good inhibitory effect on tumor blood vessels. The combined delivery strategy proposed enriches the current family of cancer biological therapies and could be further explored with different combinations of miRNA and drugs.

However, to achieve the therapeutic effects of miRNA and drugs *in vivo*, hurdles remained for effective drug delivery. The homologous targeting strategy we adopted took the advantage of the highly specific interactions of acceptors and receptors expressed on the cell membranes. With the aid of the CCM coating on the nanoparticles, our successful preparation of m-PPDCNPs, achieved at a cellular level to the extent of homologous cancer cell adhesion, targets the tumor in organs with a highly specific enrichment of CCM expression of immune-escape molecules to reduce the m-PPDCNPs via mononuclear macrophages and clear other immune cells. Thus, a sufficient number of therapeutic agents were directed to the lesions instead of being degraded or being off-target, as demonstrated by the biodistribution, which was highly specific to the tumor site instead of preferential accumulative organs such as the liver. We also verified the long-term retention of m-PPDCNPs in tumor sites after the gradual clearing of m-PPDCNPs in other sites. This advantage can ensure the specific delivery of miRNA and reduce the toxic and side effects on other organs. We believe that the homologous targeting is a preferred strategy to be involved in the nanoparticle-derived drug-delivery systems for miRNA and other drugs. In clinical practice, CCM can be obtained by biopsy puncture, a routine procedure for the diagnosis of malignant tumors. The tumor tissue removed from biopsy can be cultured and the cell membrane extracted as a carrier for homologous targeted delivery of nanoparticles.

We screened a variety of feasible schemes to obtain the polymeric nanoparticles, and finally obtained a protocol that maximized the simultaneous loading of Dox and miR-190-Cy7. The key strategy is the incorporation of 2% DC-chol into the PLGA-b-PEG. With PPDCNPs, the encapsulation efficiency of Dox reached 46%, while that of miR-190-Cy7 achieved 88%. This facile strategy effectively solves the inefficiency in miRNA loading of the PLGA-b-PEG system and provides an excellent nanodelivery platform for combination therapy. As a clinically approved agent, the PLGA polymer is generally considered safe, which is a major advantage over other biological materials with evident toxicity.[51–53] To the best of our knowledge, this system achieved the highest miRNA loading efficiency among all PLGA-based delivery systems to date. This facile manufacturing process ensures the low polydispersity, stability, and non-toxicity of the nanoparticles obtained, which implies an optimistic translational value.

Tumors are difficult to treat for a variety of reasons, mainly because tumor cells not only can escape immune attack by various means but also can develop drug resistance through genetic mutations and other methods. New research has found that common chemotherapy drugs can promote the spread of cancer, especially breast cancer.[65,67,68] Although our experimental results showed that the combination of miR-190 and Dox increased the sensitivity of HCT116 to Dox, the mechanism of this component remains unclear. miR-190 restrains Smad2/4 as the key transcription factors of the TGF-β signaling pathway in colon cancer.[19,69] Therefore, we have reason to believe that miR-190 can also help inhibit metastasis when Dox promotes the metastasis of cancer cells. We will further explore the mechanism of this aspect in future studies.

In conclusion, we developed miRNA-190-Cy7 and Dox co-encapsulated nanoparticles, m-PPDCNPs, with no biotoxicity, high loading efficiency, outstanding targeting, and precision guidance on the tumor. In the nanodelivery system of m-PPDCNPs, the efficient co-loading of Dox and miR-190 was achieved, the encapsulation efficiency of Dox reaching 46% while that of miR-190-Cy7 achieved 88%. With the homologous targeting property of CCM, the efficient uptake of CRC cells and high enrichment of tumor tissue were realized. We verified that the release of miR-190 by m-PPDCNPs inhibited the production of the key factor VEGF and thus inhibited tumor angiogenesis in multiple molecular, cellular, and histological

dimensions. At the same time, we verified that miR-190 increased the sensitivity of colon cancer cell HCT116 to Dox at the cell and tissue levels, significantly enhanced the apoptosis of cells, and realized the combination therapy. Incorporating biomedical imaging, m-PPDCNPs have significant advantages in drug-delivery monitoring, tumor imaging, and vascular therapy. With diagnostic and therapeutic properties, m-PPDCNPs have successfully achieved tumor monitoring and targeted therapy. This diagnosis and treatment concept has major implications for the treatment of CRC and beyond.

MATERIALS AND METHODS

Synthesis of PPDCNPs nanomaterials

Nanoparticles were prepared using the microemulsion method. In brief, poly(vinyl alcohol) (PVA) and Dox were dissolved in RNase-free water at a final concentration of 0.5% (w/w) PVA and 1 mg/mL Dox. The copolymer PLGA-*b*-PEG (molecular weight [MW]: 10,000), 3 mg DC-chol (MW: 500.81), and 1% Span 80 were dissolved in dichloromethane (CH_2Cl_2) at a final concentration of 1.5% (w/w) complex with respect to the polymer. 15 nmol miR-190-Cy7 mixed with spermidine (N/P ratio, 15:1) was dissolved in 3 mL of Dox solution (1 mg/mL). The 3-mL Dox solution and 10-mL PVA solution prepared before were then added and vortexed for 2 min. Subsequently, the mixed solution was treated by ultrasonic in ice bath for another 5 min. Thereafter, 20 mL of PVA solution prepared was added to the mixed solution and vortexed for 2 min. The newly mixed solution was transferred to a new 50-mL flask to be treated by ultrasonication in an ice bath for another 10 min. Meanwhile, the emulsion was injected into a three-port bottle and treated for 1 min by ice bath ultrasonication, stirring at 1,000 rpm, and vacuuming for 1 h to vaporize the CH_2Cl_2. The suspension was centrifuged at 4,000 relative centrifugal force (rcf) for 15 min and the upper liquid discarded. The suspension was resuspended with water and then passed through a 200-μm microporous membrane. The suspension was filtered through filter paper and washed three times in RNase-free water at 18,000 rcf for 15 min. Finally, the sample was freeze-dried and stored at 4°C for use.

HCT116 cancer cell membrane extraction

The Plasma Membrane Protein Cell Membrane Isolation and Cell Fractionation Kit (Invent Biotechnologies, catalog no. SM-005) was used to extract cell membranes of cancer cells, in strict accordance with the manufacturer's instructions. This can achieve the 100% separation of plasma membrane and organelle and other components.

Formulation of m-PPDCNPs

Cell plasma membrane mixed with 300 μg of DSPE-PEG2000 (Nanocs, catalog no. 121109) were physically extruded through a 200-nm Track-Etch membrane (Whatman, catalog no. 800281) for ten passes. PPDCNPs were coated in the membrane vesicles by an extruder set with holder block (Avanti, catalog no. 610000) through a 200-nm Track-Etch membrane to form m-PPDCNPs.

miR-190 and miR-190-Cy7

micrON hsa-miR-190a-5p agomir was customized and synthesized by Ribobio (catalog no. miR40000458-4-5). MicrON miRNA Agomir is a specifically chemically modified miRNA agonist. miRNA Agomir has higher stability and miRNA activity in animals. miR-190-Cy7 (catalog no. miR4160328051933-4-5) was synthesized in micrON hsa-miR-190a-5p agomir, which was modified by Sulfo-Cyanine7 at the 5′ end.

Characterization

We inspected the morphology of the PPDCNPs and m-PPDCNPs nanomaterials by TEM (Tecnai T20). Dynamic light-scattering and ζ-potential measurements were conducted with m-PPDCNPs and PPDCNPs (Zetasizer Nano ZS90). Encapsulation efficiency of Dox was detected by high-performance liquid chromatography (HPLC) (Shimadzu LC-20AT). We specified a standard curve by determining the chromatographic peak of the concentration gradient of Dox. The encapsulation rates of PPDCNPs were calculated according to the standard curve. Encapsulation efficiency of miR-190-Cy7 was detected by fluorescence spectroscopy (Edinburgh FS5). By the same method, the standard curve was established according to the concentration gradient of miR-190-Cy7, and the encapsulation rate of miR-190-Cy7 in PPDCNPs was calculated by the standard curve.

In vitro Dox and miR-190-Cy7 release

PPDCNPs were dispersed in 3-mL (pH 7.4) and 3-mL (pH 6.0) PBS solutions, respectively, and transferred to a dialysis device (MW cut-off 100 kDa; Spectrum). The 50-μL osmotic solution was extracted at a predetermined interval, and the Dox peak was detected by HPLC. The release was determined according to the standard curve. The spectral peak of miR-190-Cy7 was detected by fluorescence spectroscopy, and its release was measured according to the standard curve.

Cell culture

Colon cancer cell line HCT116 was cultured in Dulbecco's modified Eagle's medium (DMEM) containing 10% fetal bovine serum (FBS), 100 units/mL penicillin, and 0.1 mg/mL streptomycin in a 5% CO_2 incubator at 37°C. Cells were washed with PBS and incubated in 0.25% trypsin containing 5 mM EDTA. DMEM containing 10% FBS was added to terminate trypsin. After centrifugation, the pallet was diluted with the medium and counted via a hemocytometer.

Luciferase silencing

We constructed a co-transfection of 4.0×10^4 HCT116 cells with 200 ng of pGL3 Firefly luciferase construct, and 20 ng of pGL3 sea kidney luciferase standardized control. HCT116-Luci was incubated with PBS, Lipo3000 transfection of miR-190-Cy7 (Lipo-miR-190-Cy7), PPDCNPs (only miR-NC), PPDCNPs (only miR-190-Cy7), and m-PPDCNPs (only miR-190-Cy7) for 24 h or 48 h to measure the luciferase activity by the Dual Luciferase Reporter Assay System (Promega, catalog no. E1910). After 24 h or 48 h, the culture medium was precipitated, 100 μL of lysis buffer was added, the cells were lysed for 15 min at room temperature, and the supernatant was centrifuged.

The expression levels of Firefly luciferase and Renilla luciferase were detected, and the ratio was calculated. The full length of the 3′ UTR region is 1,738–3,677. The region of 2,074–3,502 was cloned. VEGFA 3′ UTR part sequence of miR-190 combined: 5′-GAG ATA TTC CGT AGT ACA TAT T.

Quantitative real-time PCR

HCT116 colon cells were incubated with PBS, Lipo3000 transfection of miR-190-Cy7, PPDCNPs (only miR-NC), PPDCNPs (only miR-190-Cy7), and m-PPDCNPs (only miR-190-Cy7) for 24 h or 48 h. The total RNA of each group was extracted by an RNA extraction kit (Transgene, catalog no. ER501), and cDNA was synthesized by a reverse transcription kit (Transgene, catalog no. AH341). Primer sequences were designed and amplified using a fluorescence quantitative PCR kit (Transgene, catalog no. AQ141), and the expression level of VEGF and GADPH were detected by the Bio-Rad Opticon2 Real-Time PCR Detector (Bio-Rad).

Human VEGF RT Primers: F 5′-GCG TGC TAA TGG TGG AAA C; R 5′-CGG TGA CAT CAA AAG ATA ACC AC.

Human GAPDH RT Primers: F 5′-TGT GGG CAT CAA TGG ATT TGG; R 5′-ACA CCA TGT ATT CCG GGT CAA T.

Enzyme-linked immunosorbent assay

The quantitative determination of human VEGF concentrations in cell culture supernatant was detected by an ELISA kit (Quantikine, catalog no. DVE00), and the operation was carried out in strict accordance with the instructions.

Western blot

The total protein of cells or tissues was extracted (Transgene, catalog no. DE101). The protein concentration was detected by the protein standard, and polyacrylamide gels with different concentrations were selected according to the MW of the protein to be detected. The SDS-PAGE glue after the separation was transferred to polyvinylidene fluoride film, put into the sealing solution, and sealed at room temperature for 1 h. The blocking solution was removed and incubated in a shaking table with primary antibody at 4°C overnight, then washed for 10 min (3 times) with 1× PBS with Tween 20 (PBST), before incubation with secondary antibody in a shaker at room temperature for 2 h followed by washing for 10 min (3 times) with 1× PBST. Color substrate NBT/BCIP was added to terminate the reaction when appropriate. Antibodies were VEGF antibody (Santa Cruz Biotechnology, catalog no. sc7269), N-cadherin antibody (Santa Cruz, catalog no. sc59987), Ep-CAM antibody (Santa Cruz, catalog no. sc-25308), galectin-3 antibody (Santa Cruz, catalog no. sc32790), CD47 antibody (Abbkine, catalog no. Abp53240), β-actin mouse antibody (Transgene, catalog no. HC201), anti-mouse IgG (H + L) HRP conjugate antibody (Transgene, catalog no. HS201), and anti-rabbit IgG HRP-linked antibody (CST, catalog no. 7074s).

In vitro toxicity assays

Luc-HCT116 cells were seeded in 96-well plates (5,000 cells per well) and incubated with DMEM (containing 10% FBS) for 12 h. After sticking to the well, the medium containing nanomaterials of different dispersion concentration was replaced for 24 h. The cells were tested for CCK8 according to the manufacturer's instructions (Transgene, catalog no. FC101).

Flow cytometry

HCT116, SW480, LS174T, HeLa, HepG2, U87, NCM460, and HUVEC cell lines were seeded in 6-well plates (50,000 cells per well) and incubated in 2 mL of DMEM (containing 10% FBS) for 24 h, The cells were then incubated with the PPDCNPs or m-PPDCNPs for another 4 h, washed five times with PBS, and collected for flow cytometry detection.

Confocal laser scanning microscopy

To investigate the homologous targeting of nanoparticles, the efficiency of m-PPDCNPs uptake by the HCT116 cell line was verified by a confocal laser scanning microscope. Cancer cells were incubated with nanoparticles for 2 h. Excitation/emission wavelength of each channel was 340/488 nm for DAPI, 450/580 nm for Dox, and 740/770 nm for Cy7.

Immunohistochemistry

After the animals were sacrificed, the tumors were completely dissected and stored in 4% paraformaldehyde solution. Paraffin sectioning, H&E staining, IHC staining (CD31), and immunofluorescence staining (TUNEL and Ki67) were performed to evaluate the expression of tissue damage and tumor-related markers.

Biodistribution

The HCT116 xenograft tumor-bearing nude mouse model was established by injection of 0.2 mL of HCT116 cell suspension (5×10^5 cells) subcutaneously to mice on the dorsal right side to investigate the *in vivo* distribution of m-PPDCNPs and PPDCNPs. At different times post injection, whole-body fluorescence images were acquired by the IVIS Lumina III fluorescence imaging system (PerkinElmer). Twenty-four hours after injection, major organs and tumors were removed and imaging was performed. To quantify the accumulation of nanoparticles in tumors and organs, Living Image software was used to quantify the fluorescence intensity of each tissue.

Statistical analysis

Statistical analysis was performed using SPSS statistical package version 26.0 (IBM SPSS, New York, NY, USA). All data are presented as mean ± SD. Statistical significance was determined by $p < 0.05$ in one-way or two-way ANOVA.

DATA AVAILABILITY

The data that support the findings of this study are available from the corresponding author (Y.L.), upon reasonable request.

SUPPLEMENTAL INFORMATION

Supplemental information can be found online at https://doi.org/10.1016/j.omto.2022.12.002.

ACKNOWLEDGMENTS

This work was supported by the Development of Key Technologies and Equipment in Major Science and Technology Infrastructure in Shenzhen under grant ZDKJ20190305003, and in part by Shenzhen Science and Technology Program under grant KQTD20180412181221912 and grant JCYJ20200109140603831.

AUTHOR CONTRIBUTIONS

S.Z. and Y.L. conceived the study and designed experiments. S.Z., Z.L., D.Z., Y.Y., and X. Ma performed the experiments and analyzed the data. Z.L., S.Z., and D.Z. performed all the statistical analyses. Z.Y. and X.M. helped design and optimize the preparation of nanomaterials. S.Z., X.M., Q.R., W.C., H.S., J.X., J.Q., D.X., Z.W., J.L., and Y.L. edited and revised the manuscript and supervised the research.

DECLARATION OF INTERESTS

The authors declare no competing interests.

REFERENCES

1. Anastasiadou, E., Jacob, L.S., and Slack, F.J. (2018). Non-coding RNA networks in cancer. Nat. Rev. Cancer *18*, 5–18.
2. Wang, L., Sinnott-Armstrong, N., Wagschal, A., Wark, A.R., Camporez, J.P., Perry, R.J., Ji, F., Sohn, Y., Oh, J., Wu, S., et al. (2020). A MicroRNA linking human positive selection and metabolic disorders. Cell *183*, 684–701.e14.
3. Yoo, A.S., Sun, A.X., Li, L., Shcheglovitov, A., Portmann, T., Li, Y., Lee-Messer, C., Dolmetsch, R.E., Tsien, R.W., and Crabtree, G.R. (2011). MicroRNA-mediated conversion of human fibroblasts to neurons. Nature *476*, 228–231.
4. Rupaimoole, R., and Slack, F.J. (2017). MicroRNA therapeutics: towards a new era for the management of cancer and other diseases. Nat. Rev. Drug Discov. *16*, 203–222.
5. Zhang, Y., Chen, W., Zhang, Y., Zhang, X., Liu, Y., and Ju, H. (2020). A near-infrared photo-switched MicroRNA amplifier for precise photodynamic therapy of early-stage cancers. Angew. Chem. Int. Ed. Engl. *59*, 21454–21459.
6. Yue, R., Chen, M., and Ma, N. (2020). Dual MicroRNA-triggered drug release system for combined chemotherapy and gene therapy with logic operation. ACS Appl. Mater. Inter. *12*, 32493–32502.
7. Chen, W.Q. (2015). Cancer statistics: updated cancer burden in China Preface. Chin. J Cancer Res *27*, 1.
8. Bozzetti, F., Doci, R., Bignami, P., Morabito, A., and Gennari, L. (1987). Patterns of failure following surgical resection of colorectal-cancer liver metastases - rationale for a multimodal approach. Ann. Surg. *205*, 264–270.
9. McQuade, R.M., Stojanovska, V., Bornstein, J.C., and Nurgali, K. (2017). Colorectal cancer chemotherapy: the evolution of treatment and new approaches. Curr. Med. Chem. *24*, 1537–1557.
10. Effenberger-Neidnicht, K., and Schobert, R. (2011). Combinatorial effects of thymoquinone on the anti-cancer activity of doxorubicin. Cancer Chemother. Pharmacol. *67*, 867–874.
11. Jayson, G.C., Kerbel, R., Ellis, L.M., and Harris, A.L. (2016). Antiangiogenic therapy in oncology: current status and future directions. Lancet *388*, 518–529.
12. Tsujii, M., Kawano, S., Tsuji, S., Sawaoka, H., Hori, M., and DuBois, R.N. (1998). Cyclooxygenase regulates angiogenesis induced by colon cancer cells. Cell *93*, 705–716.
13. Apte, R.S., Chen, D.S., and Ferrara, N. (2019). VEGF in signaling and disease: beyond discovery and development. Cell *176*, 1248–1264.
14. Lichtenberger, B.M., Tan, P.K., Niederleithner, H., Ferrara, N., Petzelbauer, P., and Sibilia, M. (2010). Autocrine VEGF signaling synergizes with EGFR in tumor cells to promote epithelial cancer development. Cell *140*, 268–279.
15. Boumahdi, S., and de Sauvage, F.J. (2020). The great escape: tumour cell plasticity in resistance to targeted therapy. Nat. Rev. Drug Discov. *19*, 39–56.
16. Miyazaki, S., Kikuchi, H., Iino, I., Uehara, T., Setoguchi, T., Fujita, T., Hiramatsu, Y., Ohta, M., Kamiya, K., Kitagawa, K., et al. (2014). Anti-VEGF antibody therapy induces tumor hypoxia and stanniocalcin 2 expression and potentiates growth of human colon cancer xenografts. Int. J. Cancer *135*, 295–307.
17. Cacheux, W., Boisserie, T., Staudacher, L., Vignaux, O., Dousset, B., Soubrane, O., Terris, B., Mateus, C., Chaussade, S., and Goldwasser, F. (2008). Reversible tumor growth acceleration following bevacizumab interruption in metastatic colorectal cancer patients scheduled for surgery. Ann. Oncol. *19*, 1659–1661.
18. Pàez-Ribes, M., Allen, E., Hudock, J., Takeda, T., Okuyama, H., Viñals, F., Inoue, M., Bergers, G., Hanahan, D., and Casanovas, O. (2009). Antiangiogenic therapy elicits malignant progression of tumors to increased local invasion and distant metastasis. Cancer Cell *15*, 220–231.
19. Hao, Y., Yang, J., Yin, S., Zhang, H., Fan, Y., Sun, C., Gu, J., and Xi, J.J. (2014). The synergistic regulation of VEGF-mediated angiogenesis through miR-190 and target genes. RNA *20*, 1328–1336.
20. van Staalduinen, J., Baker, D., ten Dijke, P., and van Dam, H. (2018). Epithelial-mesenchymal-transition-inducing transcription factors: new targets for tackling chemoresistance in cancer? Oncogene *37*, 6195–6211.
21. Zheng, X., Carstens, J.L., Kim, J., Scheible, M., Kaye, J., Sugimoto, H., Wu, C.C., LeBleu, V.S., and Kalluri, R. (2015). Epithelial-to-mesenchymal transition is dispensable for metastasis but induces chemoresistance in pancreatic cancer. Nature *527*, 525–530.
22. Vaughan, H.J., Green, J.J., and Tzeng, S.Y. (2020). Cancer-targeting nanoparticles for combinatorial nucleic acid delivery. Adv. Mater. *32*, e1901081.
23. Ren, X., Zheng, R., Fang, X., Wang, X., Zhang, X., Yang, W., and Sha, X. (2016). Red blood cell membrane camouflaged magnetic nanoclusters for imaging-guided photothermal therapy. Biomaterials *92*, 13–24.
24. Guo, Y., Wang, D., Song, Q., Wu, T., Zhuang, X., Bao, Y., Kong, M., Qi, Y., Tan, S., and Zhang, Z. (2015). Erythrocyte membrane-enveloped polymeric nanoparticles as nanovaccine for induction of antitumor immunity against melanoma. ACS Nano *9*, 6918–6933.
25. Jun, Y., Tang, Z., Luo, C., Jiang, B., Li, X., Tao, M., Gu, H., Liu, L., Zhang, Z., Sun, S., et al. (2020). Leukocyte-mediated combined targeted chemo and gene therapy for esophageal cancer. ACS Appl. Mater. Inter. *12*, 47330–47341.
26. Ning, P., Huang, L., Bao, Y., Fu, Y., Xu, C., Shen, Y., Zhou, X., Wen, X., Cheng, Y., and Qin, Y. (2020). Portfolio targeting strategy to realize the assembly and membrane fusion-mediated delivery of gold nanoparticles to mitochondria for enhanced NIR photothermal therapies. Bioconjug. Chem. *31*, 2719–2725.
27. Dong, Y., Siegwart, D.J., and Anderson, D.G. (2019). Strategies, design, and chemistry in siRNA delivery systems. Adv. Drug Deliv. Rev. *144*, 133–147.
28. Xu, X., Wu, J., Liu, Y., Saw, P.E., Tao, W., Yu, M., Zope, H., Si, M., Victorious, A., Rasmussen, J., et al. (2017). Multifunctional envelope-type siRNA delivery nanoparticle platform for prostate cancer therapy. ACS Nano *11*, 2618–2627.
29. Fang, R.H., Kroll, A.V., Gao, W., and Zhang, L. (2018). Cell membrane coating nanotechnology. Adv. Mater. *30*, e1706759.
30. Kroll, A.V., Fang, R.H., and Zhang, L. (2017). Biointerfacing and applications of cell membrane-coated nanoparticles. Bioconjug. Chem. *28*, 23–32.
31. Cho, H., Gao, J., and Kwon, G.S. (2016). PEG-b-PLA micelles and PLGA-b-PEG-b-PLGA sol-gels for drug delivery. J. Control Release *240*, 191–201.
32. Devulapally, R., Foygel, K., Sekar, T.V., Willmann, J.K., and Paulmurugan, R. (2016). Gemcitabine and antisense-microRNA Co-encapsulated PLGA-PEG polymer nanoparticles for hepatocellular carcinoma therapy. ACS Appl. Mater. Inter. *8*, 33412–33422.
33. Risnayanti, C., Jang, Y.S., Lee, J., and Ahn, H.J. (2018). PLGA nanoparticles co-delivering MDR1 and BCL2 siRNA for overcoming resistance of paclitaxel and cisplatin in recurrent or advanced ovarian cancer. Sci. Rep. *8*, 7498.

34. Li, W., Qiu, J., Li, X.L., Aday, S., Zhang, J., Conley, G., Xu, J., Joseph, J., Lan, H., Langer, R., et al. (2021). BBB pathophysiology-independent delivery of siRNA in traumatic brain injury. Sci. Adv. *7*, eabd6889.

35. Hrkach, J., Von Hoff, D., Mukkaram Ali, M., Andrianova, E., Auer, J., Campbell, T., De Witt, D., Figa, M., Figueiredo, M., Horhota, A., et al. (2012). Preclinical development and clinical translation of a PSMA-targeted docetaxel nanoparticle with a differentiated pharmacological profile. Sci. Transl. Med. *4*, 128ra39.

36. Gamonet, C., Desmarets, M., Mourey, G., Biichle, S., Aupet, S., Laheurte, C., François, A., Resch, E., Bigey, F., Binda, D., et al. (2020). Processing methods and storage duration impact extracellular vesicle counts in red blood cell units. Blood Adv. *4*, 5527–5539.

37. Oldenborg, P.A., Zheleznyak, A., Fang, Y.F., Lagenaur, C.F., Gresham, H.D., and Lindberg, F.P. (2000). Role of CD47 as a marker of self on red blood cells. Science *288*, 2051–2054.

38. Casey, S.C., Tong, L., Li, Y., Do, R., Walz, S., Fitzgerald, K.N., Gouw, A.M., Baylot, V., Gütgemann, I., Eilers, M., and Felsher, D.W. (2016). MYC regulates the antitumor immune response through CD47 and PD-L1. Science *352*, 227–231.

39. Lindberg, F.P., Bullard, D.C., Caver, T.E., Gresham, H.D., Beaudet, A.L., and Brown, E.J. (1996). Decreased resistance to bacterial infection and granulocyte defects in IAP-deficient mice. Science *274*, 795–798.

40. Rodriguez, P.L., Harada, T., Christian, D.A., Pantano, D.A., Tsai, R.K., and Discher, D.E. (2013). Minimal "self" peptides that inhibit phagocytic clearance and enhance delivery of nanoparticles. Science *339*, 971–975.

41. Weiskopf, K., Ring, A.M., Ho, C.C.M., Volkmer, J.P., Levin, A.M., Volkmer, A.K., Ozkan, E., Fernhoff, N.B., van de Rijn, M., Weissman, I.L., and Garcia, K.C. (2013). Engineered SIRP alpha variants as immunotherapeutic adjuvants to anticancer antibodies. Science *341*, 88–91.

42. Suk, J.S., Xu, Q., Kim, N., Hanes, J., and Ensign, L.M. (2016). PEGylation as a strategy for improving nanoparticle-based drug and gene delivery. Adv. Drug Deliv. Rev. *99*, 28–51.

43. Inglut, C.T., Baglo, Y., Liang, B.J., Cheema, Y., Stabile, J., Woodworth, G.F., and Huang, H.C. (2019). Systematic evaluation of light-activatable biohybrids for anti-glioma photodynamic therapy. J. Clin. Med. *8*, 1269.

44. Xiong, Y., Zhao, Y., Miao, L., Lin, C.M., and Huang, L. (2016). Co-delivery of polymeric metformin and cisplatin by self-assembled core-membrane nanoparticles to treat non-small cell lung cancer. J. Control Release *244*, 63–73.

45. Devulapally, R., Sekar, N.M., Sekar, T.V., Foygel, K., Massoud, T.F., Willmann, J.K., and Paulmurugan, R. (2015). Polymer nanoparticles mediated codelivery of AntimiR-10b and AntimiR-21 for achieving triple negative breast cancer therapy. ACS Nano *9*, 2290–2302.

46. Zheng, M., Yue, C., Ma, Y., Gong, P., Zhao, P., Zheng, C., Sheng, Z., Zhang, P., Wang, Z., and Cai, L. (2013). Single-step assembly of DOX/ICG loaded lipid-polymer nanoparticles for highly effective chemo-photothermal combination therapy. ACS Nano *7*, 2056–2067.

47. Wang, Z., Ju, Y., Ali, Z., Yin, H., Sheng, F., Lin, J., Wang, B., and Hou, Y. (2019). Near-infrared light and tumor microenvironment dual responsive size-switchable nanocapsules for multimodal tumor theranostics. Nat. Commun. *10*, 4418.

48. Urano, Y., Asanuma, D., Hama, Y., Koyama, Y., Barrett, T., Kamiya, M., Nagano, T., Watanabe, T., Hasegawa, A., Choyke, P.L., and Kobayashi, H. (2009). Selective molecular imaging of viable cancer cells with pH-activatable fluorescence probes. Nat. Med. *15*, 104–109.

49. Webb, B.A., Chimenti, M., Jacobson, M.P., and Barber, D.L. (2011). Dysregulated pH: a perfect storm for cancer progression. Nat. Rev. Cancer *11*, 671–677.

50. Prajakta, D., Ratnesh, J., Chandan, K., Suresh, S., Grace, S., Meera, V., and Vandana, P. (2009). Curcumin loaded pH-sensitive nanoparticles for the treatment of colon cancer. J. Biomed. Nanotechnol. *5*, 445–455.

51. Jain, R.A. (2000). The manufacturing techniques of various drug loaded biodegradable poly(lactide-co-glycolide) (PLGA) devices. Biomaterials *21*, 2475–2490.

52. Lück, M., Pistel, K.F., Li, Y.X., Blunk, T., Müller, R.H., and Kissel, T. (1998). Plasma protein adsorption on biodegradable microspheres consisting of poly(D, L-lactide-co-glycolide), poly(L-lactide) or ABA triblock copolymers containing poly(oxyethylene). Influence of production method and polymer composition. J. Control Release *55*, 107–120.

53. Shive, M.S., and Anderson, J.M. (1997). Biodegradation and biocompatibility of PLA and PLGA microspheres. Adv. Drug Deliv. Rev. *28*, 5–24.

54. Charbe, N.B., Amnerkar, N.D., Ramesh, B., Tambuwala, M.M., Bakshi, H.A., Aljabali, A.A.A., Khadse, S.C., Satheeshkumar, R., Satija, S., Metha, M., et al. (2020). Small interfering RNA for cancer treatment: overcoming hurdles in delivery. Acta Pharm. Sin. B *10*, 2075–2109.

55. Hafez, I.M., Maurer, N., and Cullis, P.R. (2001). On the mechanism whereby cationic lipids promote intracellular delivery of polynucleic acids. Gene Ther. *8*, 1188–1196.

56. Verma, A., and Stellacci, F. (2010). Effect of surface properties on nanoparticle-cell interactions. Small *6*, 12–21.

57. Jin, J., Krishnamachary, B., Barnett, J.D., Chatterjee, S., Chang, D., Mironchik, Y., Wildes, F., Jaffee, E.M., Nimmagadda, S., and Bhujwalla, Z.M. (2019). Human cancer cell membrane-coated biomimetic nanoparticles reduce fibroblast-mediated invasion and metastasis and induce T-cells. ACS Appl. Mater. Inter. *11*, 7850–7861.

58. Cui, L., Gouw, A.M., LaGory, E.L., Guo, S., Attarwala, N., Tang, Y., Qi, J., Chen, Y.S., Gao, Z., Casey, K.M., et al. (2021). Mitochondrial copper depletion suppresses triple-negative breast cancer in mice. Nat. Biotechnol. *39*, 357–367.

59. Sun, H., Su, J., Meng, Q., Yin, Q., Chen, L., Gu, W., Zhang, P., Zhang, Z., Yu, H., Wang, S., and Li, Y. (2016). Cancer-cell-biomimetic nanoparticles for targeted therapy of homotypic tumors. Adv. Mater. *28*, 9581–9588.

60. Baek, K.H., Zaslavsky, A., Lynch, R.C., Britt, C., Okada, Y., Siarey, R.J., Lensch, M.W., Park, I.H., Yoon, S.S., Minami, T., et al. (2009). Down's syndrome suppression of tumour growth and the role of the calcineurin inhibitor DSCR1. Nature *459*, 1126–1130.

61. Carmeliet, P. (2005). Angiogenesis in life, disease and medicine. Nature *438*, 932–936.

62. Chin, L., Tam, A., Pomerantz, J., Wong, M., Holash, J., Bardeesy, N., Shen, Q., O'Hagan, R., Pantginis, J., Zhou, H., et al. (1999). Essential role for oncogenic Ras in tumour maintenance. Nature *400*, 468–472.

63. Ferrara, N., and Kerbel, R.S. (2005). Angiogenesis as a therapeutic target. Nature *438*, 967–974.

64. Abdullah, C.S., Alam, S., Aishwarya, R., Miriyala, S., Bhuiyan, M.A.N., Panchatcharam, M., Pattillo, C.B., Orr, A.W., Sadoshima, J., Hill, J.A., and Bhuiyan, M.S. (2019). Doxorubicin-induced cardiomyopathy associated with inhibition of autophagic degradation process and defects in mitochondrial respiration. Sci. Rep. *9*, 2002.

65. Keklikoglou, I., Cianciaruso, C., Guc, E., Squadrito, M.L., Spring, L.M., Tazzyman, S., Lambein, L., Poissonnier, A., Ferraro, G.B., Baer, C., et al. (2019). Chemotherapy elicits pro-metastatic extracellular vesicles in breast cancer models. Nat. Cell Biol. *21*, 190–202.

66. Derynck, R., Turley, S.J., and Akhurst, R.J. (2021). TGFbeta biology in cancer progression and immunotherapy. Nat. Rev. Clin. Oncol. *18*, 9–34.

67. Huang, P., Ouyang, D.J., Chang, S., Li, M.Y., Li, L., Li, Q.Y., Zeng, R., Zou, Q.Y., Su, J., Zhao, P., et al. (2018). Chemotherapy-driven increases in the CDKN1A/PTN/PTPRZ1 axis promote chemoresistance by activating the NF-kappaB pathway in breast cancer cells. Cell Commun. Signal. *16*, 92.

68. Xiang, L., and Semenza, G.L. (2019). Hypoxia-inducible factors promote breast cancer stem cell specification and maintenance in response to hypoxia or cytotoxic chemotherapy. Adv. Cancer Res. *141*, 175–212.

69. Yu, Y., and Cao, X.C. (2019). miR-190-5p in human diseases. Cancer Cell Int. *19*, 257.

OMTO, Volume *28*

Supplemental information

A cancer cell membrane coated, doxorubicin and microRNA co-encapsulated nanoplatform for colorectal cancer theranostics

Sihao Zhu, Ziyuan Li, Dongye Zheng, Yue Yu, Jing Xiang, Xiao Ma, Dongqing Xu, Jiajun Qiu, Ziyu Yang, Zhiyi Wang, Jun Li, Hongfang Sun, Weiqiang Chen, Xiangxi Meng, Yanye Lu, and Qiushi Ren

Table S1 Identification of optimal method to load Dox in PEGylated-PLGA nanoparticles

No.	PLGA-b-PEG (mg/mL)	Drug phase	Organic solvents	Surfactant for emulsification	Dox (mg)	Mean size[a] (nm)	PDI[a]	ζ potential[a]	Encapsulation efficiency (%)
1	20	Water	DCM	0.5% Span, 0.5% PVA	2	108.4	0.145	-15.6	27.46
2	20	Oil	DCM	0.5% Span, 0.5% PVA	2	120.3	0.173	-14.7	25.26
3	10	Water	DCM	0.5% Span, 0.5% PVA	2	53.7	0.196	-15.2	24.4
4	20	Oil	THF	0.5% PVA	2	245.4	0.282	-19.7	10.78
5	20	Water	DCM	—	2	545.6	0.396	-14.3	38.11
6	20	Water	DCM	0.5% span, 0.5% PVA	3	113.7	0.164	-13.2	29
**7**	**10**	**Water**	**DCM**	**0.5% span, 0.5% PVA**	**3**	**54.83**	**0.171**	**-13.8**	**54.51**
8	20	Oil	DCM	0.5% Span, 0.5% PVA	3	96.4	0.247	-12.9	30.77
9	20	Oil	THF	0.5% PVA	3	274.2	0.375	-15.6	40.52
10	20	Water	DCM	—	3	543.7	0.631	-19.3	58.74

[a] Note: average of three DLS measurements.

Table S2 Identification of optimal Method To Co-load miR-190-Cy7 and Dox in PPDCNPs.

No.	PLGA-b-PEG (mg/mL)	DC-chol (mg/mL)	Surfactant for emulsification	Drug concentration		Mean size (nm)[a]	PDI[a]	ζ potential[a]	Encapsulation efficiency (%)	
				Dox (mg)	miR-190-Cy7 (nmol)				Dox	miR-190-Cy7
1	10	0	0.5% Span 0.5% PVA	3	15	58.63	0.16	-23.23	49.60	19.34
2	10	0.1	0.5% Span 0.5% PVA	3	15	68.06	0.158	-7.75	47.22	80.65
3	**10**	**0.2**	**0.5% Span 0.5% PVA**	**3**	**15**	**90.43**	**0.173**	**2.45**	**46.01**	**88.33**
4	10	0.3	0.5% Span 0.5% PVA	3	15	103.76	0.193	7.64	21.88	90.39
5	10	0.5	0.5% Span 0.5% PVA	3	15	151.13	0.214	15.48	9.40	91.71
6	10	1.0	0.5% Span 0.5% PVA	3	15	193.56	0.232	27.14	2.50	93.22

[a] Note: average of three DLS measurements.

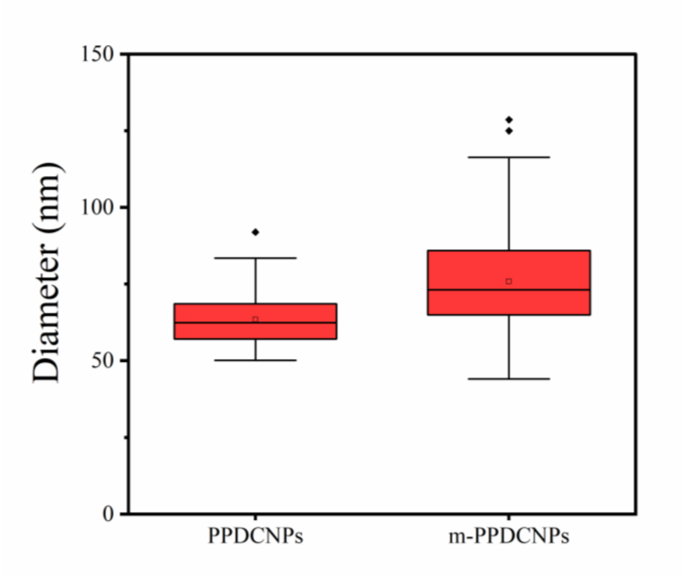

Figure S1 Size distribution of around 100 nanoparticles.

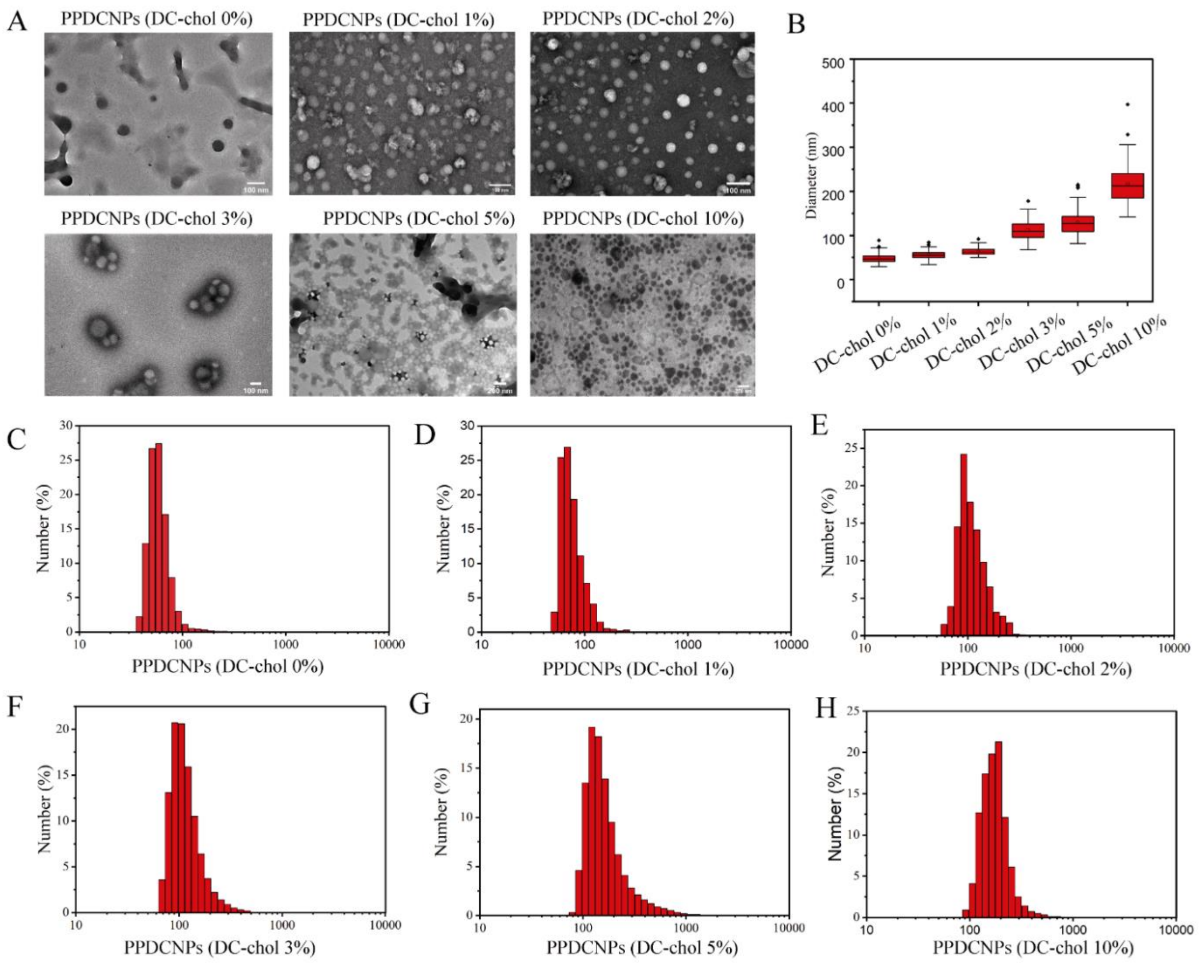

Figure S2 TEM images and hydrodynamic sizes of PPDCNPs.

(A) TEM image of PPDCNPs with different concentrations of DC-chol. (B) Size distribution of around 100 nanoparticles chosen from A. (C-F) Hydrodynamic size of miR-190 and Dox encapsulated PPDCNPs measured by dynamic light scattering.

Note: The figure on first row third column was also used in manuscript Figure 2D.

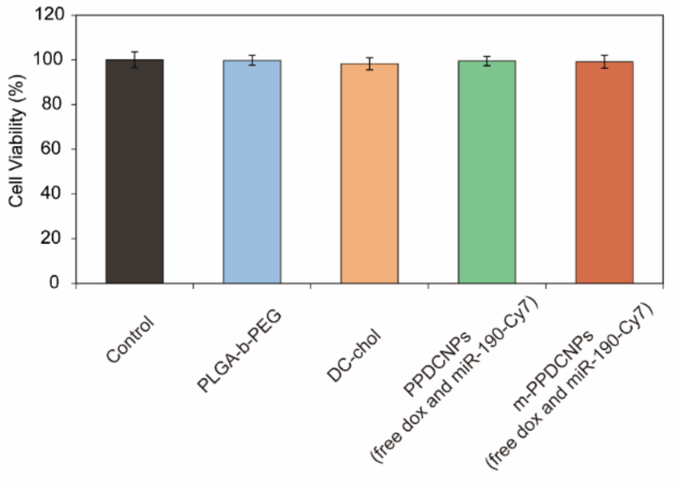

Figure S3 Cytotoxicity analysis of m-PPDCNPs.

Proliferation profile of HCT116 cells incubated with PBS, 10 mg/mL PLGA-b-PEG, 0.2mg/ml DC-chol, 10mg/ml PPDCNPs (free miR-190-Cy7 and Dox), 10 mg/mL m-PPDCNPs (free miR-190-Cy7 and Dox) for 24 h to analyze the Cytotoxicity of m-PPDCNPs.

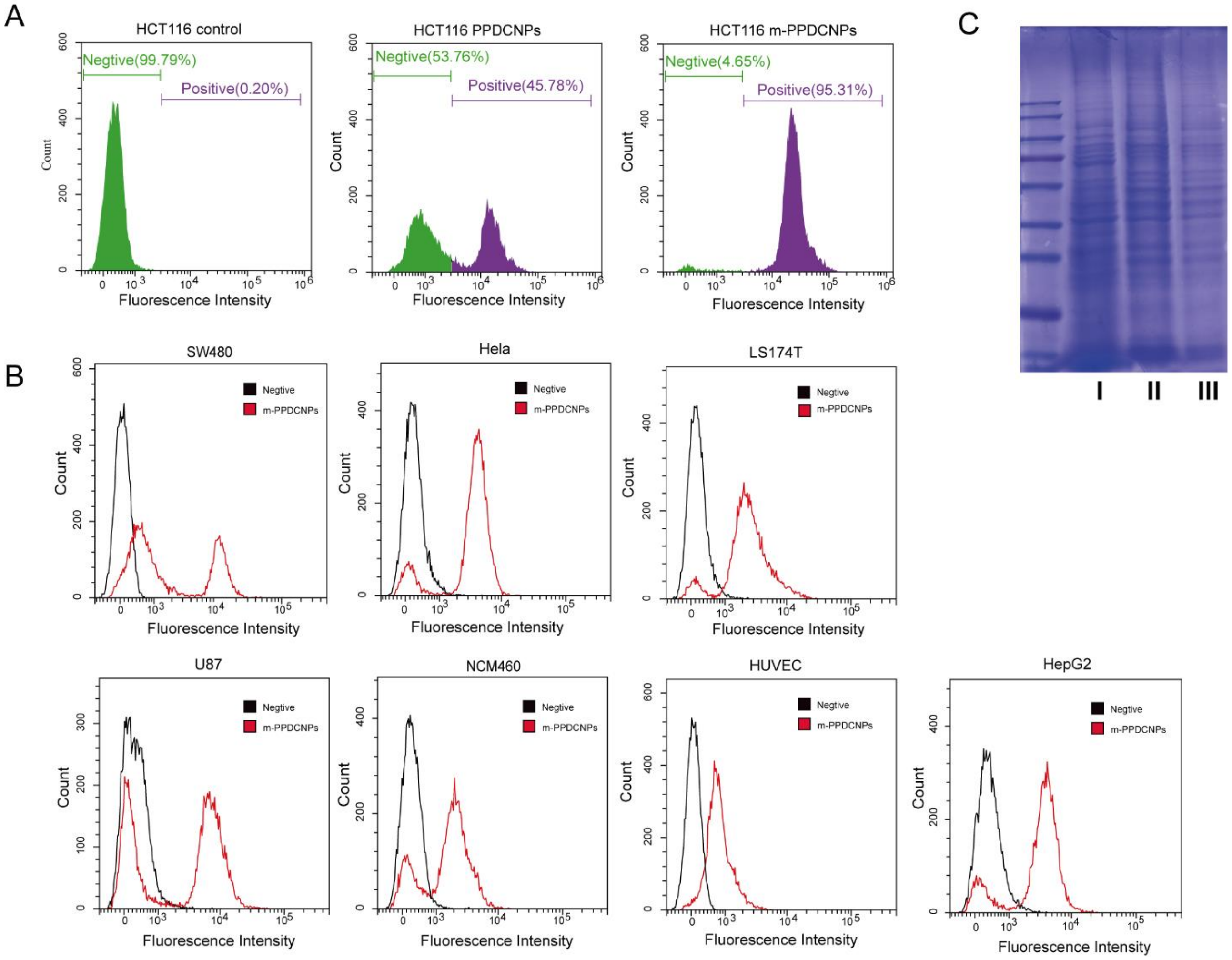

Figure S4 *In vitro* CRC-targeting ability of the miR-190-Cy7 and Dox loaded m-PPDCNPs.

(A) Flow cytometric profiles of HCT116 incubated with PPDCNPs and m-PPDCNPs. (B) Flow cytometric profiles of the seven cell lines SW480, LS174T, Hela, HepG2, U87, NCM460, and HUVEC upon 2 h incubation with m-PPDCNPs. (C) SDS-PAGE method to analyze protein composition. I: HCT116 Cancer cell total protein, II: cancer cell membrane protein, III: m-PPDCNPs.

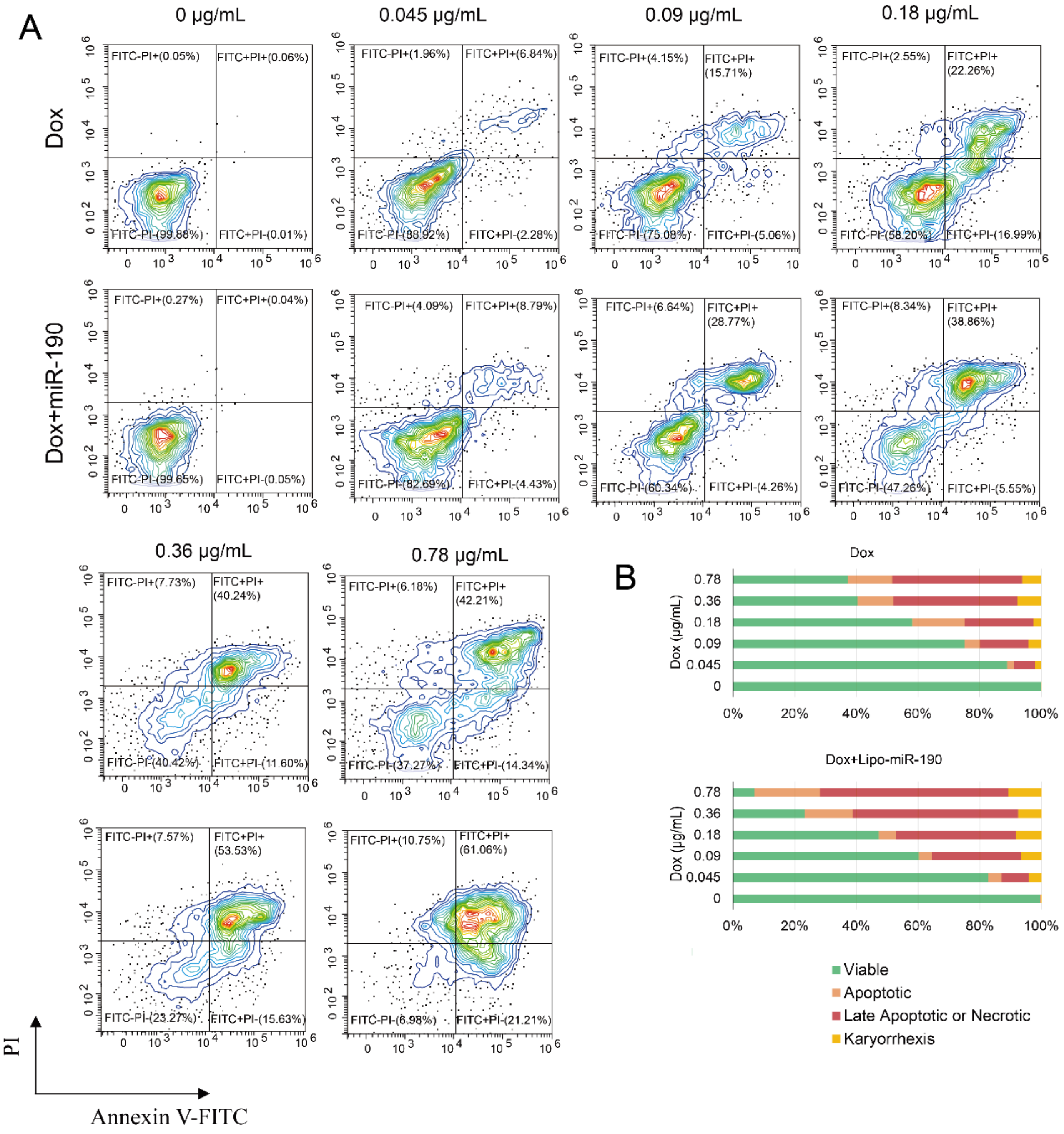

Figure S5 Flow cytometry analysis of apoptosis of HCT116 cells using Annexin V-FITC and PI staining.

(A) Apoptosis of HCT116 cells treated with Lipo3000-miR-190 and Dox with gradient concentration(0 μg/mL, 0.045 μg/mL, 0.09 μg/mL, 0.18 μg/mL, 0.36 μg/mL, 0.78 μg/mL) by flow cytometry analysis using Annexin V-FITC and PI staining, H_2O_2-treated cells were used as a positive control. (B) Apoptosis analysis of HCT116 cells by FACs.

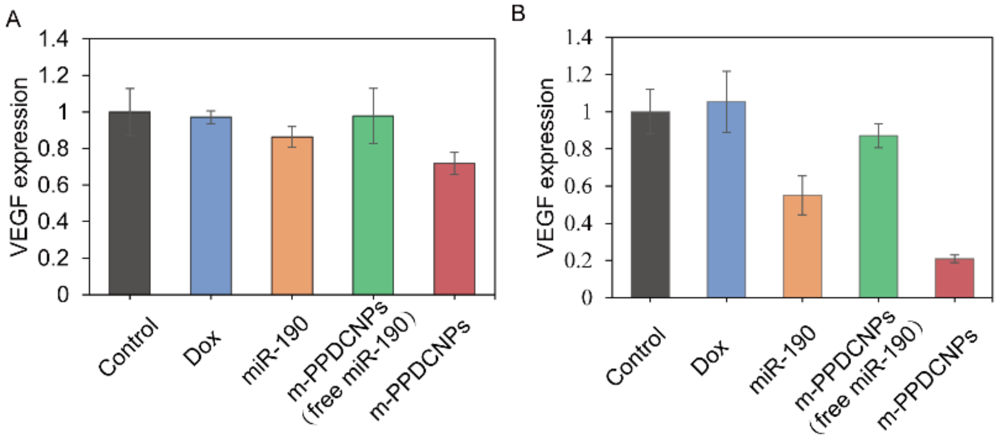

Figure S6 Western blot analysis and quantification of VEGF expression in the HCT116 tumor tissue after systemic treatment by PBS, miR-190, Dox, m-PPDCNPs (free miR-190-Cy7) and m-PPDCNPs.

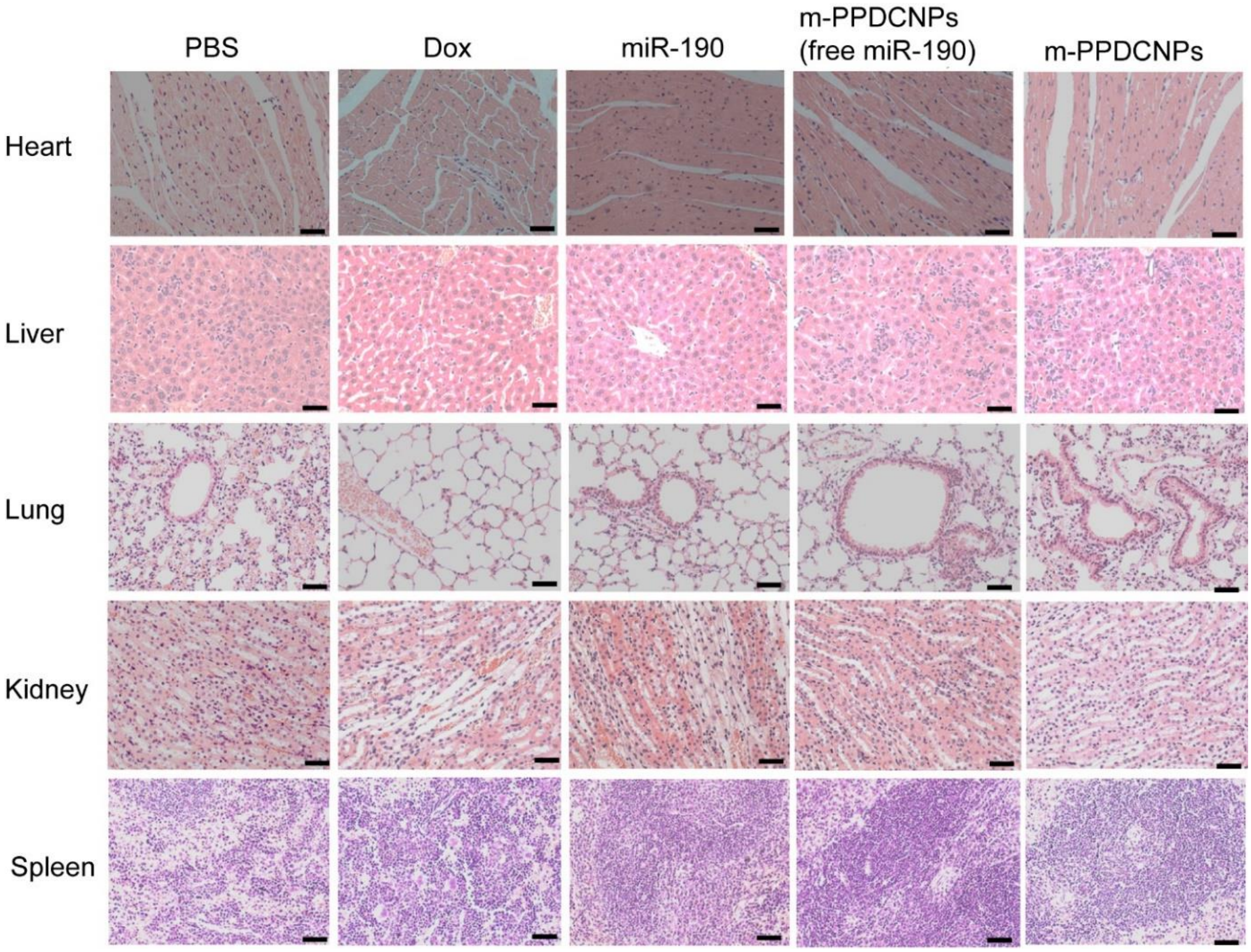

Figure S7 In vivo safety of m-PPDCNPs assessed by histopathological analysis.

Histological sections of the major organs (including heart, liver, lung, kidney, spleen) of mice intravenously injected with PBS, Dox, miR-190, m-PPDNPs (free miR-190) and m-PPDNPs for evaluating the safety and systemic toxicity in vivo of each group after 22 treatment. Hematoxylin-eosin; Scale bar: 50 μm.

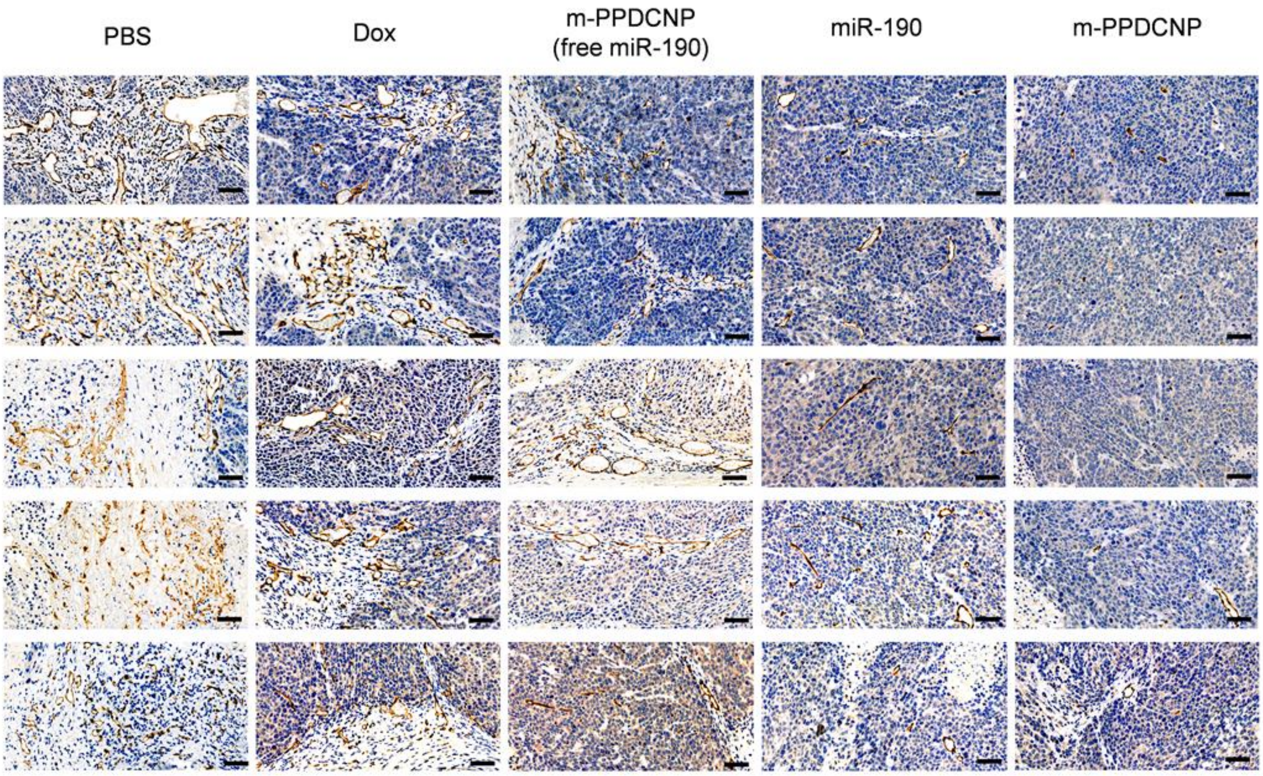

Figure S8 Immunohistochemistry CD31 staining sections of tumor.

Immunohistochemistry CD31 staining sections of primary tumors of HCT116 xenograft tumor-bearing nude mice after treatment by PBS, miR-190, and Dox and m-PPDCNPs (free miR-190-Cy7). Scale bar: 50 μm.

Note: The figures on first line were also used in manuscript Figure 6G.

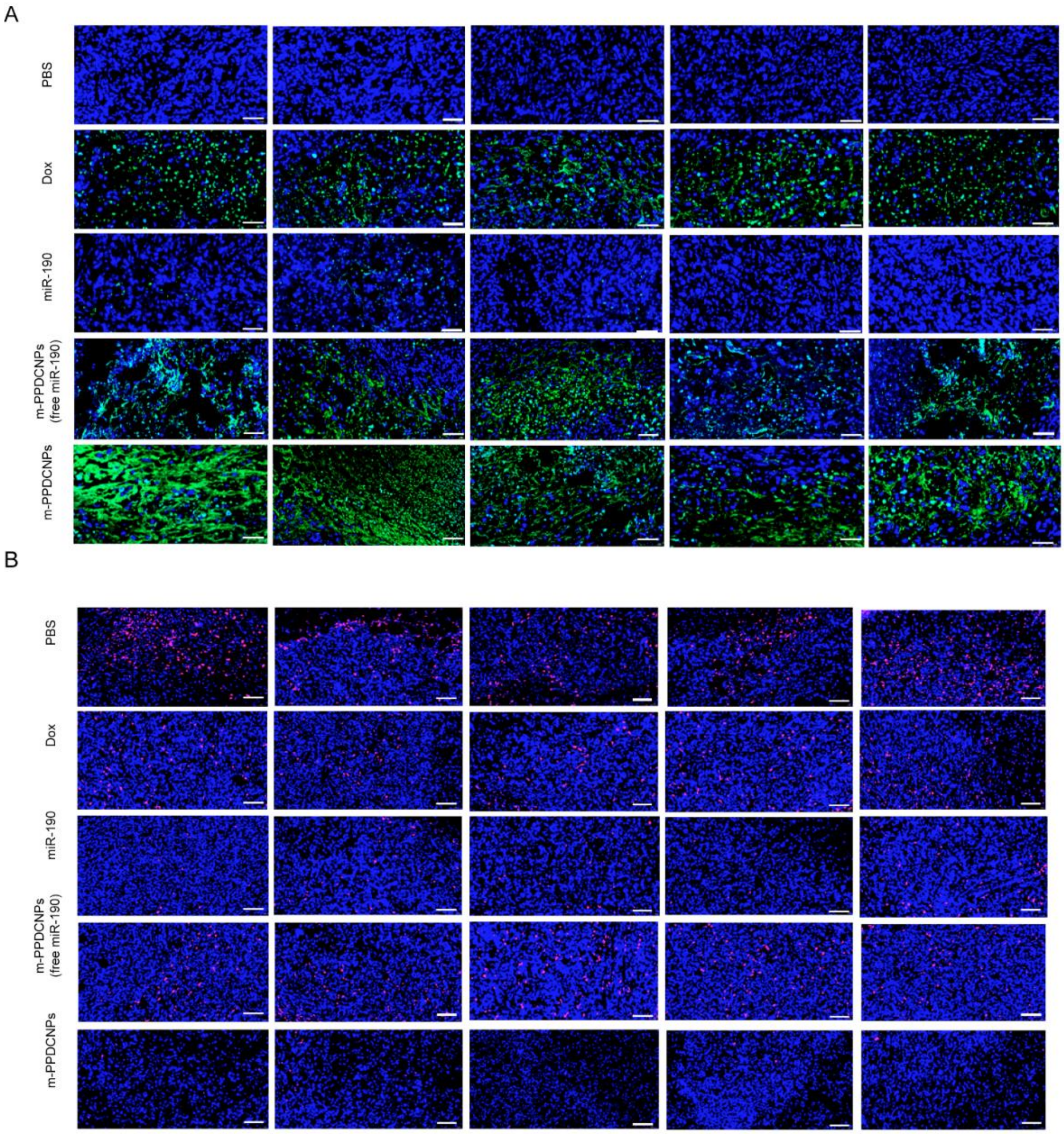

Figure S9 Immunofluorescent staining of TUNEL or KI67 expression in tumor.

Immunofluorescent staining of TUNEL(A) or KI67 (B) expression in tumor tissue sections of HCT116 xenograft tumor-bearing nude mice after treatment by PBS, miR-190, Dox and m-PPDCNPs (free miR-190-Cy7). Scale bar: 50 μm.

Note: The first column of (A) and (B) were also used in manuscript Figure 6H.
